# The unique genomic landscape surrounding the *EPSPS* gene in glyphosate resistant *Amaranthus palmeri*: a repetitive path to resistance

**DOI:** 10.1186/s12864-016-3336-4

**Published:** 2017-01-17

**Authors:** William T. Molin, Alice A. Wright, Amy Lawton-Rauh, Christopher A. Saski

**Affiliations:** 1 United States Department of Agriculture, Crop Production Systems Research Unit, Stoneville, MS USA; 2 Department of Genetics and Biochemistry, Clemson University, Clemson, SC USA; 3 Institute of Translational Genomics, Genomics and Computational Biology Lab, Clemson University, Clemson, SC USA

**Keywords:** Weedy species, Herbicide resistance, *Amaranthus palmeri*, *EPSPS cassette*, Transposable elements, Adaptive evolution

## Abstract

**Background:**

The expanding number and global distributions of herbicide resistant weedy species threaten food, fuel, fiber and bioproduct sustainability and agroecosystem longevity. Amongst the most competitive weeds, *Amaranthus palmeri* S. Wats has rapidly evolved resistance to glyphosate primarily through massive amplification and insertion of the 5-enolpyruvylshikimate-3-phosphate synthase (*EPSPS*) gene across the genome. Increased *EPSPS* gene copy numbers results in higher titers of the EPSPS enzyme, the target of glyphosate, and confers resistance to glyphosate treatment. To understand the genomic unit and mechanism of *EPSPS* gene copy number proliferation, we developed and used a bacterial artificial chromosome (BAC) library from a highly resistant biotype to sequence the local genomic landscape flanking the *EPSPS* gene.

**Results:**

By sequencing overlapping BACs, a 297 kb sequence was generated, hereafter referred to as the “*EPSPS cassette*.” This region included several putative genes, dense clusters of tandem and inverted repeats, putative helitron and autonomous replication sequences, and regulatory elements. Whole genome shotgun sequencing (WGS) of two biotypes exhibiting high and no resistance to glyphosate was performed to compare genomic representation across the *EPSPS cassette*. Mapping of sequences for both biotypes to the reference *EPSPS cassette* revealed significant differences in upstream and downstream sequences relative to *EPSPS* with regard to both repetitive units and coding content between these biotypes. The differences in sequence may have resulted from a compounded-building mechanism such as repetitive transpositional events. The association of putative helitron sequences with the cassette suggests a possible amplification and distribution mechanism. Flow cytometry revealed that the *EPSPS cassette* added measurable genomic content.

**Conclusions:**

The adoption of glyphosate resistant cropping systems in major crops such as corn, soybean, cotton and canola coupled with excessive use of glyphosate herbicide has led to evolved glyphosate resistance in several important weeds. In *Amaranthus palmeri*, the amplification of the *EPSPS cassette*, characterized by a complex array of repetitive elements and putative helitron sequences, suggests an adaptive structural genomic mechanism that drives amplification and distribution around the genome. The added genomic content not found in glyphosate sensitive plants may be driving evolution through genome expansion.

**Electronic supplementary material:**

The online version of this article (doi:10.1186/s12864-016-3336-4) contains supplementary material, which is available to authorized users.

## Background

Weedy species are a continuous threat to crop and pasture land, requiring constant mitigation to minimize yield losses, protect commodity quality, and prevent infestations in subsequent years. Estimates of crop losses due to weeds worldwide in wheat, maize and cotton production for 2001 to 2003 were 7.7, 10.5, and 8.6%, respectively [1]. Although numerous management practices are available to farmers, such as tillage and cultivation, the use of herbicides remains the most effective and preferred method in minimizing weed interference. In past decades, herbicide-based strategies have performed well in weed management. However, in recent years several weeds have evolved resistance to some of the most widely used herbicides, such as glyphosate, and have become a serious threat to crop production in the last decade. Glyphosate resistant Palmer amaranth (*Amaranthus palmeri* S. Wats) alone has increased production costs of corn by $2 to $35 per acre, $0 to $100 per acre in cotton, and $6 to $42 per acre in soybean [2]. *A. palmeri* is a fast growing, highly competitive, yield-reducing weed of row crops that must be controlled throughout the crop development cycle to minimize losses. With the introduction of glyphosate resistant crops in 1996 (GR cropping systems), farmers gained an effective tool to control seedling *A. palmeri.* GR cropping systems permitted repeated applications throughout the crop season with excellent crop safety while avoiding tilling practices that reduce soil longevity and cause erosion. The broad species spectrum and efficacy of control with glyphosate contributed to the expansion of no- and reduced-tillage production systems [2].

The first reports of glyphosate resistant *A. palmeri* emerged nearly ten years after the introduction of GR cropping systems, in 2005, in Georgia and North Carolina [3, 4]. Since then, reports of glyphosate resistant *A. palmeri* have been confirmed in 25 states [4]; it continues to spread across the southern states, into the Ohio Valley and into the northeastern states as far north as Pennsylvania and New Jersey. It is not clear whether glyphosate resistance across all of these regions is derived from single or independent adaptive events. Moreover, several of these glyphosate resistant *A. palmeri* populations also have resistance to other herbicides, most commonly acetolactate synthase inhibitors [4].

Several molecular mechanisms facilitate resistance to glyphosate, including target-site mutation, target-site gene duplication, active vacuole sequestration, limited cellular uptake, and a rapid necrosis response [5–7]. Target-site gene duplication was recently discovered to be the primary genetic mechanism underlying glyphosate resistance in *A. palmeri*. The increase in gene copies of the 5-enolpyruvylshikimate-3-phosphate synthase (*EPSPS*) gene, which encodes the protein targeted by glyphosate, was positively correlated with increased *EPSPS* cDNA expression and translated EPSPS protein levels [5]. The presence of elevated EPSPS protein levels conferred resistance. Increased gene copy number is also a known genetic mechanism of acquired herbicide resistance in *Kochia scoparia* from Montana [8] and from Kansas, Colorado, North Dakota, and South Dakota [9], in *A. tuberculatus* (waterhemp) from Illinois, Kansas, Missouri and Nebraska [10], in *Lolium perenne* ssp. *multiflorum* from Arkansas [11], and in *Bromus diandrus* from Australia [12]. This mode of resistance has also been observed to be acquired by interspecific hybridization between *A. palmeri* and *A. spinosus* [13].

Fluorescence in situ hybridization (FISH) analysis of somatic metaphase chromosomes and interphase nuclei in the cells of glyphosate resistant plants with the *EPSPS* gene as a probe showed fluorescence signals distributed throughout the *A. palmeri* genome, providing visual evidence for the existence of multiple copies of the *EPSPS* gene [5]. Gaines et al. [6] estimated that somewhere between 30 and 50 *EPSPS* genome copies were required for *A. palmeri* to survive glyphosate applications between 0.5 and 1.0 kg/ha. In contrast, the genomic organization of amplified *EPSPS* in *K. scoparia* was different. The *EPSPS* gene was localized to one end of a pair of homologous chromosomes and was organized as a tandem array of ten copies [14], rather than seemingly random copy distribution throughout the genome. In both cases, the mechanism directing the gene amplification was not elucidated. To define the landscape of the amplified unit, its size (length), associated regulatory components, and putative features that drive amplification of *EPSPS* under selective pressure, Gaines and coworkers constructed a fosmid library from genomic DNA of a resistant plant from Mississippi, identified and sequenced *EPSPS* containing clones [15]. This study generated a consensus sequence of ~30 kb, including the *EPSPS* which comprised about 10 kb. An additional 1.5 kb of sequence upstream and 20 kb of sequence downstream were amplified along with the *EPSPS* locus. Analysis of the additional sequence revealed that the *EPSPS* gene was bordered by miniature inverted-repeat transposable elements (MITEs), and a putative Activator (Ac) transposase was located further downstream on the 3′ end. There was no sequence divergence amongst any of the fosmids at either end, suggesting that the entire 30 kb was amplified. Further probing of the fosmid library did not identify additional candidate clones suitable for extending the genomic segment, justifying the need for a different approach to obtain the entire *EPSPS cassette*.

The large size of the amplified sequence (30 kb) and the apparent association with MITEs and a putative Activator transposase supported a transposon-mediated duplication process, but the presence of introns opposed a retrotransposon-based mechanism [15]. This was further supported by the lack of sequence polymorphisms in the amplified *EPSPS* gene [15]. Based on these data, it seems plausible that the *EPSPS* gene amplification may fit a replicative transposition mechanism in which the copy number of the cassette increases because replicated progeny of the element are transposed to another chromosomal site [16]. FISH analysis indicated the presence of the cassette on multiple chromosomes [5], supporting a model that the transposition of *EPSPS* involved insertions into chromosomes other than the one from which it originated. Jugulam et al. [14] proposed that the amplification in *K. scoparia* arose from unequal crossover. However, in *K. scoparia* the *EPSPS* gene copies appear in tandem on one chromosome suggesting a mechanism that is different in scale and scope from that in *A. palmeri* [14].

Gene duplication and amplification can be facilitated by several repeat-based mechanisms such as transposable elements, DNA palindromes, and a less understood class of repeats, DNA helitrons, which have an increasing evidence base of functional presence among plants and animals, that includes an extra-chromosomal mode of amplification [17]. DNA palindromes have been characterized as sites of genome instability and are associated with chromosomal alterations such as deletions and translocations, in addition to gene amplification in prokaryotic and eukaryotic cells [18]. These repeat types tend to form complex DNA secondary structure and cruciforms, which are the targets for many architectural and regulatory proteins. Non-random repeat complexes have been found in the vicinity of breakpoint junctions and promoter regions, as well as the sites of replication initiation [19–21] and are implicated in adverse biological functions such as increased drug resistance and the advancement of tumorigenesis in cancer systems. Helitrons are a more recently discovered class of DNA transposons that transpose as a rolling circle replicon [17]. Distinguishing features of helitrons include the lack of terminal repeats, lack of duplication of host insertion sites, inclusion of palindromes of variable sequence length, and precise insertion into the host genome [17].

The present study focused on determining the genomic organization and evolutionary machinery underlying *EPSPS* amplification, including elucidation of the replicative genome mechanism responsible for local repetitive, coding, and regulatory content. Further analysis included how these features potentially function or interact during selection for glyphosate resistance to mediate elevated resistance in *A. palmeri*.

## Results

### *A. palmeri* BAC library construction, recruitment of *EPSPS* BACs, tile path selection, and sequencing

**Fig. 1:**
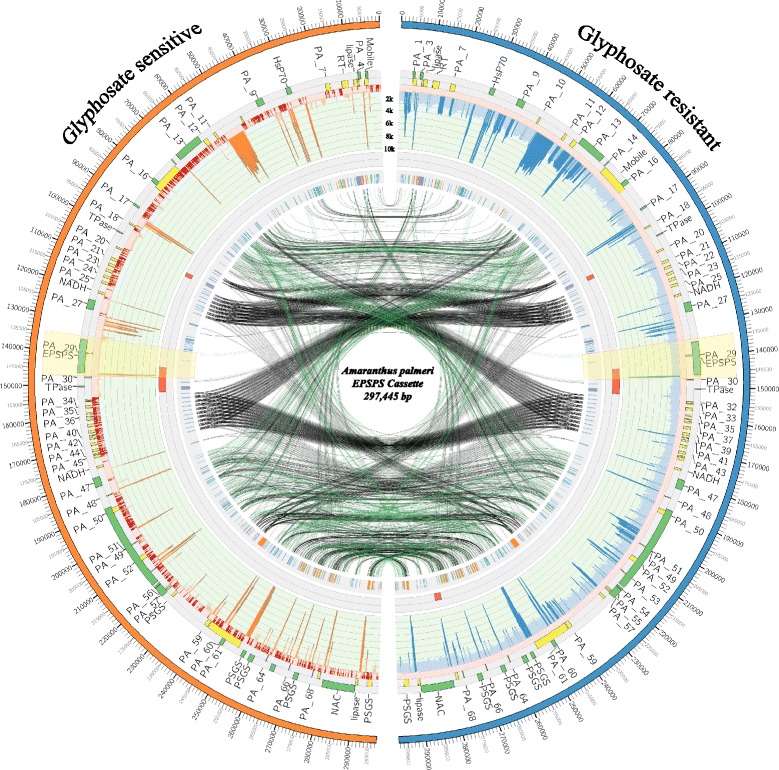
The glyphosate resistant *EPSPS* locus and surrounding genomic landscape; highlighted in yellow is the *EPSPS* gene. The outer ideograms (*blue* is glyphosate resistant biotype and orange is glyphosate sensitive) are situated to depict the assembled 297,445 bp as a self-alignment. The outermost track is the gene annotation where the gene histograms that are transcribed in the clockwise direction are plotted in green in the outward direction, and gene histograms transcribed counterclockwise are plotted in *yellow* on the internal axis. The line graphs (*orange and blue*) represent aligned resistant and susceptible WGS reads reported as coverage depths. The light blue and light orange colors represent regions covered with up to 2,000 reads, and dark blue and dark orange mark regions covered by greater than 2,001 reads. Dark red glyphs indicate bases with “0” coverage. The inner track with red only histograms depicts predicted helitron sequences with LCV values of at least ‘3’, and the innermost histogram track displays predicted repetitive elements. The internal links connect repetitive regions by self-alignment of the molecule; black links are direct repeats and green are inverted in orientation

BAC libraries are critical tools for genomic dissection and they serve as the primary templates for targeted sequencing to facilitate accurate reconstruction of the genomic architecture around genes, especially in large repetitive plant genomes [22–24]. Here, we constructed the first high quality BAC library from a glyphosate resistant biotype of *A. palmeri* that consists of 36,864 clones with an average insert size of 110 kb. Based on an estimated genome size of 938 Mbp (2C) for the *A. palmeri* genome [25], the BAC resources represent approximately 8.6 genome equivalents. The BAC library was screened for clones containing the *EPSPS* locus. A total of 680 clones (~2%) were identified as positive to the *EPSPS* probe, suggesting high representation in the genome and the BAC library (Additional file 1: Figure S1A). Because the large number of positively identified BACs likely correlates with multiple genomic copies of *EPSPS*, we fingerprinted 192 BACs to assess overlap (see Methods) and determine a tile path of representative BACs. Under stringent fingerprint assembly parameters, 186 BACs clustered into a single contig, and a tile path of 4 representative BAC clones was selected for individual sequencing on the Illumina MiSeq platform and as a pool on the Pacific Biosystems (PacBio) RSII platform. The Illumina-only assemblies resulted in hundreds to thousands of small-unordered contigs, suggesting a high repetitive content. We were unable to produce an assembly with contiguity, and efforts were redirected to the long read single molecule PacBio dataset. A total of 65,733 raw reads were collected and filtered for length that resulted in a total of 57,957 reads with lengths between 1,000 bp and 31 kb, with a mean read length of 6.1 kb (Accession SAMN06007219). A *de novo* assembly was performed that resulted in a single consensus sequence (see Methods) that amassed 228,387 bp. A subsequent round of DNA hybridization was performed with overgo probes that were designed from the terminal sequences that were not represented elsewhere within the 228 kb genome segment, which resulted in approximately 100 hits per BAC filter (Additional file 1: Figure S1B). Four additional extension BACs were sequenced to produce a final assembly length of 297,445 bp (Fig. 1). The *A. palmeri* BAC library (APC_Ba) and filter set is publically available at www.genome.clemson.edu.

### The genomic landscape of the *EPSPS cassette*

The genomic architecture of the *EPSPS* locus includes a complex configuration of repetitive DNA arrays interspersed with genes and gene fragments, which together are hallmark signatures of amplification and mobilization of genomic segments. Approximately 140 kb of genomic sequence was captured at both 5′ and 3′ of the *EPSPS* locus (Fig. 1). Gene prediction algorithms identified *EPSPS* and 71 putative genes (27 genes 5′ and 44 genes 3′ of the *EPSPS* locus), (Fig. 1 and Additional file 2: Table S1). Twenty-five of the putative genes produced a BLAST result against either the SwissProt or TrEMBL databases (Additional file 2: Table S1). Of the putative genes that returned a BLAST result, several contain signature functional themes underlying defense or response to stress, DNA mobility, sequestration, hormonal control, suppressor of gene silencing, and heat shock. Two gene copies with transposase domains, which resemble the *Ricesleeper*1 gene, were identified that immediately flank both sides the *EPSPS* locus. The copy downstream of the *EPSPS* is the putative Ac transposase identified in the fosmid study [15]. These genes are members of an angiosperm-specific gene family that have transposase domains [26]. Additional putative genes identified include six gene copies with plant-mobile domains (2 of which have transit localization sequences that target the mitochondria) organized at both the 5′ and 3′ ends of the cassette; a single copy of a viral-derived reverse transcriptase; 2 phospholipase gene copies that are likely involved in defense signaling [27]; a heat shock cognate 70 kDa protein (*HSC70*); a NAC domain containing gene; a ubiquitin-like protease (*Ulp*1); and a tandem cluster containing 6 copies of suppressor of gene silencing 3 (*SGS*3) genes located ~77 kb downstream from the *EPSPS* gene (Fig. 1 and Additional file 2: Table S1). These suppressors of gene silencing genes have been shown to be essential in post-transcriptional gene silencing and natural virus resistance [28]. However, *SGS3* is in excess of 600 amino acids in length [29] and the copies in the cassette are less than half that, with the longest at 240 amino acids, indicating that even if expressed, they may not generate a functional enzyme. Moreover, their truncated presence, arrangement as a cluster, and possible fractionation, may be signatures of genomic amplification events. Six of the 72 genes (including the *EPSPS* gene) contain chloroplast localization signals, 9 contain mitochondrial localization signals, and 3 contain signals for secretion (Fig. 1 and Additional file 2: Table S1). Transcriptional activity of three of the putative genes was confirmed for the reverse transcriptase (PA_6), *HSC70* (PA_8), and the NAC domain containing protein (PA_70) by quantitative PCR in both *EPSPS* sensitive and resistant biotypes (for copy number data, see Additional file 3: Table S2). Both the *EPSPS* and the reverse transcriptase showed an increase in gene expression in the resistant plants, however the expression for the *HSC70* and the NAC domain containing protein were more variable. The reverse transcriptase showed the greatest increase in transcription with 268.9 fold greater expression in the resistant plant (Table 1). Transcripts of the reverse transcriptase were barely detectable in the sensitive plant. Expression of the *HSC70* and the NAC domain containing protein was variable among replicates (Table 1 and data not shown), however these are stress response genes and differences in expression between R and S biotypes may not be apparent in plants not subjected to abiotic or biotic stressors.

**Table 1:** Transcript quantification (qRTPCR) for the EPSPS and three putative genes

	EPSPS	NAC	HSC70	Reverse Transcriptase
R replicate 1	6.44 +/− 0.318	0.0101 +/− 0.00054	4.27 +/− 0.222	2.14 +/− 0.112
R replicate 2	12.18 +/− 0.179	0.0128 +/− 0.00064	0.69 +/− 0.104	9.62 +/− 0.397
S replicate 1	0.126 +/− 0.0046	0.00172 +/− 0.0000684	0.977 +/− 0.0711	0.00746 +/− 0.00605
S replicate 2	0.501 +/− 0.022	0.00362 +/− 0.000148	2.33 +/− 0.159	Not detected
Fold change (R1/S1)	51.1	5.9	4.4	286.9
Fold change (R2/S2)	24.3	3.5	0.3	N/A

To assess DNA structure, a self-alignment of the *EPSPS cassette* was performed and it revealed a complex repetitive landscape composed of an assortment of transposons and helitrons intermixed among DNA palindromes and simple repeats (Fig. 1, Table 2, and Additional file 4: Table S3). The internal links shown in Fig. 1 represent sequences with self-similarity that compose a pattern of forward (black links) and reverse (green links) palindromic sequences dispersed as short tandem arrays flanking the *EPSPS* gene. Moreover, there are larger blocks of direct and inverted repeat sequences situated outside of the palindromic repeat arrays, as depicted in the self-alignment dot plot (Additional file 5: Figure S2). The arrangement of repeat architecture and content suggest the potential for inter-molecular recombination.

**Table 2:** Repeat characterization of the EPSPS amplicon

Class I Transposons (copy and paste)	
LTR Retroposons	
LTR	18
LTR/Copia	17
LTR/Gypsy	3
Non-LTR Retroposons	
LINE/L1	2
LINE/RTE-BovB	1
SINE	14
SINE/tRNA-RTE	1
Low_complexity	16
Class II Transposons (cut and paste type)	
DNA	
DNA/CMC-EnSpm	3
DNA/hAT-Ac	11
DNA/hAT-Tip100	12
DNA/MULE-MuDR	2
MITE	
DNA/TcMar-Stowaway	5
Repeats	
Simple_repeat	192
Helitrons (LCV ≥ 3)	
helitron (rolling circle)	3

The *EPSPS cassette* was subject to a repeat characterization (see Methods) to identify known repetitive elements (Table 2 and Additional file 4: Table S3). A total of 300 repeat sequences were identified in the 297 kb genomic segment, with the most abundant repeat class representing simple repeats (192). The remaining 108 repeats are composed of Class I Transposons (72 total; copy and paste type), Class II Transposons (33 total; cut and paste type), and a novel class of repeats associated with genome reshuffling and gene amplification called helitrons (3 total) (Table 2, Fig. 1, Additional file 4: Table S3 and Additional file 6: Table S4). The head of helitron 3 spans the 3′ end of the *EPSPS* gene, indicating a possible vehicle of amplification for the *EPSPS* gene (Fig. 1).

### Whole genome sequencing (WGS) of Sensitive (S) and Resistant (R) biotypes

To quantify and characterize genomic content and structure of the *EPSPS cassette* in lines with contrasting glyphosate resistant phenotypes, we performed whole genome shotgun sequencing of both glyphosate sensitive and resistant biotypes from the same geographical area (Accessions: SAMN06007217, SAMN06007218). We collected approximately 51 million read pairs for the resistant biotype and 85 million read pairs (approximately 68% more data) for the sensitive biotype (Additional file 7: Table S5). A total of 4,017,582 reads uniquely mapped to the *EPSPS cassette* from the resistant biotype, and only 1,313,550 mapped from the sensitive biotype accounting for 3.93% and 0.77% of all reads from the resistant and sensitive biotype, respectively (Fig. 1, Additional file 7: Table S5). Sequence reads from the resistant biotype mapped concordantly, for the most part, and completely covered the entire *EPSPS* reference interval; however, extensive differences were observed in comparison to the sensitive biotype coverage. The average coverage per base of the resistant biotype is 1,650 reads per base and 519 reads per base for sensitive biotype, respectively. All 72 putative genes and repeat sequences present in the *EPSPS cassette* were deeply covered by the resistant biotype whole-genome shotgun sequence reads. Across the entire *EPSPS cassette* there was greater depth of coverage in the resistant biotype compared to the sensitive, indicating that the entire sequence was amplified in the resistant biotype. That the coverage in the resistant biotype did not taper off at either end of the sequence also indicates that the ends of the cassette have not been reached and that there is additional amplified sequence.

Mapping reads from the sensitive biotype to the *EPSPS cassette* revealed numerous gaps (both coding and intergenic), suggesting that the cassette sequence is not contiguous in the sensitive biotype (Fig. 1). The sensitive biotype differed extensively in coverage when mapped to the *EPSPS* reference interval with 49,091 bp in the reference with 0 read depth, and 57,572 bp with only 1 or 2 reads mapping, which accounts for a total of 106,663 bases (36% of the cassette) with very low or no coverage (Fig. 1). The mean exon coverage was 1,336 and 109 reads per base, for the resistant and the sensitive biotype, respectively. Moreover, the sensitive biotype had a total of 83 exons as parts of 37 gene bodies with interrupted coverage (bases with ‘0’ reads mapping). Of the 37 genes, 8 genes were completely missing where each exon was not covered at all in the S biotype, and these were mainly uncharacterized protein sequences and one copy of the *SGS*3 suppressor of gene silencing gene (PA_65). Genes with interrupted exonic coverage were also mostly uncharacterized protein sequences as well as the peptide chain release factor subunit (PA_10), RE1-silencing transcription factor (PA_13), Plant mobile protein, 5 of the 6 copies of the *SGS3* cluster, Ulp protease, and the NAC transcription factor (PA_70). Interestingly, gene bodies completely intact in the sensitive WGS dataset include the Plant Mobile cluster (PA_2 and PA_3), both copies of the phospholipase-like protein (PA_5 and PA_71) that flank the *EPSPS* gene, the reverse transcriptase (PA_6), the heat shock protein (PA_8), both transposase genes (PA_19 and PA_31) that flank the *EPSPS* gene, the glutamate synthase (PA_26), the replication protein (PA_27), the *EPSPS* gene (PA_28), and one copy of the *SGS*3 gene (PA_58) (Fig. 1).

**Fig. 2:**
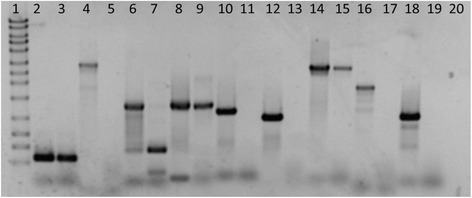
PCR amplifications of specific sites within the cassette for glyphosate resistant and susceptible *A. palmeri*. Lane 1: 1 kb ladder with band sizes of 10, 8, 6, 5, 4, 3, 2.5, 2, 1.5, 1, 0.7, 0.5, and 0.3 kb. Even lanes are the resistant *A. palmeri* and odd lanes are the sensitive. Lanes 2–3: primers binding within the putative HSC70 gene. Lanes 4–5: primers downstream of the HSC70. Lanes 6–7: primers upstream of the first putative rice sleeper gene. Lanes 8–9: primers within the putative rice sleeper gene. Lanes 10–11: primers immediately downstream of the first putative *ricesleeper* gene. Lanes 12–13: primers immediately upstream of *EPSPS*. Lanes 14–15: primers within *EPSPS*. Lanes 16–17: primers downstream of the second putative *ricesleeper* gene. Lanes 18–19: primers 20 kb upstream of the end of known cassette. Lane 20: negative control

The presence of gaps in the sensitive plant was confirmed by PCR analysis (Fig. 2). Primers were designed to amplify regions of the cassette that appeared contiguous in the sensitive biotype and the sequence flanking those regions. Contiguous regions included the putative *HSC70* gene (lanes 2 and 3), the putative rice sleeper gene (lanes 8 and 9), and *EPSPS* (lanes 14 and 15). The remaining lanes are reactions that assay the intervening or adjacent sequence. In these, a band is present for the resistant plant but absent or much smaller in the sensitive. These data support the lack of contiguity in the sensitive biotype predicted by the gaps in the map.

To assess and gauge consistency and robustness of the WGS data, we determined coverage statistics of 5 gene sequences encoded distal to the *EPSPS cassette* (Additional file 8: Table S6). Alignment of the WGS datasets to the well characterized acetolactate synthase (ALS) gene, a known low copy gene with monogenic inheritance [30] produced an average of 46% more reads in the sensitive WGS dataset. Four other genes assessed for coverage that are present as single copies as confirmed in Southern blots (Lawton-Rauh, unpublished), include a transmembrane nine super-family, a DEAD-box RNA helicase, a subtilisin-like protease, and a monothiol glutaredoxin. Alignments of the relative sensitive and resistant biotype genomic sequence reads also resulted in the expected read depth coverage (~40% more sensitive reads than resistant reads), which serves as an excellent control that the dataset is robust and predictable in other genomic regions of known copy number (Additional file 8: Table S6). Based on knowledge of expected coverage of single-copy gene sequence (Additional file 8: Table S6), we found that the intact *SGS*3 gene in the sensitive biotype was present as ~8 copies, both transposases (PA_31 and PA_19) were present in ~4 copies, the replication proteins (PA_27) and the heat shock gene (PA_8) were present as ~2 copies, and the *EPSPS* (PA_28), glutamate synthase (PA_26), and the phospholipase genes (PA_5 and PA_71) were present as 1-2 copies.

A comparison of the repetitive structure between the sensitive and resistant biotypes revealed that coverage of the 3 putative helitrons, including the helitron sequence that is predicted to encompass part of the *EPSPS* locus, is present with contiguous coverage in the sensitive biotype (Additional file 9: Table S7). The coverage differences ranged from 54 to 89% fold differences in the resistant biotype (Additional file 9: Table S7). All of the 298 annotated repeat sequences depicted in the innermost histogram tracks in Fig. 1, and listed in Additional file 4: Table S3, were identified with deep coverage in the resistant biotype WGS sequences. However, the sensitive biotype differed extensively, with 54 repeats not detected, and 5 repeats partially covered, leaving a total of 72 repeats with significant differences between the biotypes (Additional file 4: Table S3). The non-represented repeats were mainly Simple repeats (44), Non-LTR retrotransposons (6), Low complexity sequence (3), and Class II Transposons (2). Of the 238 intact repeat sequences in the sensitive WGS data, the most abundant (>7000 read coverage) were the DNA/TcMar-Stowaway class II transposon, simple repeats, LTR retrotransposons, and *copia* elements. The direct repeat clusters that form the palindromes flanking the *EPSPS* gene (~110 kb-120 kb and 152 kb-168 kb) were deeply covered in the resistant biotype WGS. However, these clusters were either not present or present in extremely low abundance in the sensitive biotype WGS (Fig. 1).

The sensitive biotype exhibited a high degree of difference from the reference in both coding and repetitive content. A single nucleotide polymorphism analysis (SNP) was performed to assess the level of single nucleotide variation between the resistant and sensitive biotypes relative to each other and the *EPSPS* reference sequence. We observed a total of 1,624 SNP differences between the sensitive and resistant biotypes within the *EPSPS cassette* (Additional file 10: Figure S3 red and blue glyphs). Many of these changes could be simple differences in genetic background, but the observation of an abundance of SNP polymorphisms and lack of repetitive sequence in the sensitive biotype suggests that these differences were due to assembly and amplification of the *EPSPS cassette* in the resistant plants.

### Syntenic comparison of the *EPSPS cassette* with *Amaranthus tuberculatus, Amaranthus hypochondriacus,* and *Beta vulgaris*

To assess structural homology with closely related genomes, the *EPSPS cassette* was aligned to the latest draft genome assemblies of *Amaranthus tuberculatus* [31], *Amaranthus hypochondriacus* [32], and *Beta vulgaris* [33] (Additional file 11: Figure S4). By assessing alignments with match identities ≥ 60% and match lengths ≥ 100 bp, the highest degree of collinearity was observed between the *EPSPS cassette* and *A. hypochondriacus* with a total of 776 genomic scaffolds that sum to a total of ~788 kb (Additional file 12: Table S9 and Additional file 11: Figure S4A). Alignment to the skim sequence assembly of *A. tuberculatus* also produced a significant number of scaffolds (135) with similarity to the *EPSPS cassette* (Additional file 11: Figure S4B and Additional file 12: Table S9). However, this draft version of the genome is quite fragmented with over 100 k unanchored scaffolds that lack long range contiguity with N_50_ scaffold lengths of ~42 kb [32], so determining macro/micro-synteny with the *EPSPS* remains challenging. It was confirmed *in silico* that the *A. hypochondriacus* assembly contains a single copy of the *EPSPS* gene on scaffold kn127846.1 (Additional file 13: Figure S5A) and the exon structure is collinear with high identity to that of the *EPSPS cassette* (Additional file 13: Figure S5B). Likely because of the short length of this scaffold (~45 kb), it was not possible to identify other syntenic genes flanking the *EPSPS* gene. When aligning just the predicted genes to the *A. hypochondriacus* assembly, there is a high degree of similarity and content. The alignment revealed a tandem gene cluster of plant mobile genes with copies in alternate orientations; multiple copies and fragments of the Reverse Transcriptase gene (PA_6), multiple copies and fragments of the heat shock gene (PA_8), a tandem cluster of the Suppressors of Gene Silencing (SGS3) gene, one copy of the transposase (PA_31), the Ulp (PA_69), and NAC domain gene (PA_70) (Additional file 13: Figure S5A). Due to the lack of completeness and contiguity, it was difficult to perform an accurate assessment of repeat structure between *A. hypochondriacus* and the *EPSPS cassette.* By arranging the alignment layout to order scaffolds relative to the *EPSPS cassette* (Additional file 11: Figure S4A), gaps and collinearity that resembles that of an *A. palmeri* sensitive biotype were observed (Fig. 1). The published *A. tuberculatus* draft genome [31] was produced by assembling a small amount of pyrosequence data and has an assembly length of only 4.3 Mbp (~15 k contigs); but it was still possible to anchor a total of 135 scaffolds with a length similar to our *EPSPS cassette*, 293,880 bp (Additional file 11: Figure S4B). The length of the *A. tuberculatus* matches summed to 293,880 bp, which resemble the *EPSPS cassette* length (Additional file 11: Figure S4B). However, we were unable to identify an *EPSPS* containing scaffold, likely due to the lack of coverage, and abandoned further detailed analysis. Alignment to the closely related *Beta vulgaris* genome assembly revealed similarity with several of the repetitive elements, with a total 738 scaffolds (737 of which are not placed in the main assembly) that summed to ~15.8 M bp (Additional file 11: Figure S4C and Additional file 12: Table S9). Alignment of the genes yielded a similar number of complete and fragmented reverse transcriptase and heat shock genes (PA_6 and PA_8, respectively), a single copy of the *EPSPS* gene, and only 3 copies of the SGS3 genes encoded distally in the genome (Additional file 13: Figure S5A). An examination of the *EPSPS* containing scaffold revealed that only the reverse transcriptase gene (PA_6) gene is encoded in the predicted order of the *EPSPS cassette* (Additional file 13: Figure S5B).

### Genome size differences as a result of *EPSPS cassette* amplification

The size of the *EPSPS cassette,* depth of coverage, and hypothesis that the complete unit is amplified raised the possibility that a measurable increase in total genomic DNA had occurred. This increase is detectable by flow cytometry. One sensitive biotype with no historical presentation of glyphosate was used as a standard and 4 resistant-confirmed biotypes with *EPSPS* copy numbers ranging from 77 to 106 (Table 3) were used. Four replicates of each biotype were used. The unselected control genome size was determined to be 0.82 pg/2C with a standard deviation of ±0.005 (Additional file 14: Table S9). Next, the resistant biotypes with *EPSPS* copy numbers of 77 and 79 were sized at 0.88 (6.8% genome increase) and 0.85 pg/2C, respectively (3.5% genome increase). Ninety-two copies measured at 0.87 pg/2C (5.75% genome increase), and the highest copy number, 106 copies, yielded the largest estimation of genomic content, 0.93 pg/2C (11.8% genome increase) (Table 3 and Additional file 14: Table S9). Hence, genome size correlates with *EPSPS* copy number. Based on these findings, this approach can be used to estimate the size of the *EPSPS cassette*. For example, between 23 and 31 extra megabases of genomic DNA were observed (Table 3), that correlates with increasing *EPSPS* gene copy number (77–106 copies); and the data suggests that the complete *EPSPS cassette* is amplified, so it is estimated that the complete cassette could range from 600 to 1,100 kbp in size (Table 3), assuming 1 pg/2C = 1Mbp. An average of the estimated lengths gives 625 kb, with a deviation of ± sti, so the cassette length is somewhere between 313 kb and 937 kb.

**Table 3:** Genome size estimations based on flow cytometry of genotypes with known EPSPS cassette copies, and estimation of the size of the EPSPS cassette

Genotype	Sensitive (S) or Resistant (R)	Genome size estimated by FLOW (pg/2C)	EPSPS gene copy number (Relative to ALS)	% increase relative to S	Estimated genome size increase (based on ~ 300 kb cassette) (Mbp)	Estimated size of the EPSPS Cassette (Kbp) based on observed FLOW Data	Average of estimated sizes (±50%)
PI632235	S	0.82	1	N/A	N/A	N/A	
P3	R	0.88	77.09	6.82	23	600	
P4	R	0.85	79.96	3.53	24	300	625
W3	R	0.87	92.66	5.75	28	500	
P1	R	0.93	106.62	11.83	32	1,100	

## Discussion

The first case of glyphosate resistance in an *Amaranthus palmeri* population was reported in the U.S. state of Georgia, and this resistance was associated with massive amplification of the *EPSPS* gene (>100-fold increase) [5]. Fluorescence in situ hybridization (FISH) indicated that these *EPSPS* copies are distributed throughout the genome [5]. Very little is understood about the adaptive molecular mechanism(s) endowing survival of lethal herbicide applications, especially the relative role of gene copy number proliferation in either promoting or accelerating herbicide resistance in weeds. We characterized the genomic landscape surrounding the repeated *EPSPS* genome region to understand how this genome region was built before and after duplication, and the potential molecular mechanism(s) that led to high levels of amplification and migration across the genome. Understanding the structural features and components of the entire region will shed light on potential mechanisms driving this gene amplification event culminating in glyphosate resistance.

Previous studies have shown that the genomic sequence flanking the *EPSPS* gene in *A. palmeri* is rich in repeat units, making targeted nucleotide sequencing challenging and elusive with short read technology [15]. BAC libraries and hierarchical BAC sequencing approaches are still contemporary methods in dissecting large, complex genomes and for accurately resolving difficult to sequence genomic regions [34–36]. The challenges previously identified using fosmids and short read sequencing in determining the genomic landscape surrounding the *EPSPS* gene in *A. palmeri* [15] underscore the need for longer DNA molecules, e.g., BACs that can be isolated and targeted for extending our knowledge of the origin of glyphosate resistance, the *EPSPS cassette*. To characterize this region, a BAC library was prepared from glyphosate resistant *A. palmeri* plants (see Methods) and then BAC clones tiling the *EPSPS cassette* were identified and sequenced utilizing Pacific Biosciences’ single molecule technology. We also employed whole genome shotgun sequencing of both a sensitive and resistant biotype of *A. palmeri* to compare genomic composition and organization around the reference *EPSPS cassette* and to characterize signatures of resistance evolution.

The previously reported *EPSPS* segment, approximately 30 kb in length [15], was extended to 297 kb with near equal sized upstream and downstream consensus additions bordering a single copy of the *EPSPS* gene. The *EPSPS* gene was embedded as a single copy gene within a repeat dense multi-gene complex termed the *EPSPS cassette*. Interestingly, no evidence has been found to support a local tandem *EPSPS* arrangement as has been reported in herbicide resistant *K. scoparia* [14]. As the *EPSPS cassette* has only one copy of EPSPS, resistance was due to accumulation of multiple copies of the *EPSPS*-containing cassette. Despite long sequences bordering the *EPSPS* gene, there are few polymorphisms in the WGS data. This pattern supports the report by Gaines et al. [15] that the cassette was of recent origin and has not been subject to recombination. Another possible explanation is a very fast and strong selective sweep that carried the entire cassette rapidly to fixation as a full unit with recombinants across the cassette not having as high of fitness thus dropping out from the population over generations. In this alternative mechanism, the full cassette could have been maintained because the homologous ends enable the rapid amplification and insertion of the cassette into the genome. Recombination would break up this essential homologous binding and prevent efficient amplification and genome-wide proliferation of the *EPSPS* gene (and other genes within the cassette that possibly play a role in adaptation). The *EPSPS cassette* has little homology with genomic sequences from the sampled sensitive plant, indicating that the *EPSPS cassette* represents either a newly derived sequence of very recent origin or a new arrangement of existing sequences in *A. palmeri*, which in either case leads to an expansion and mechanism enabling rapid adaptation. To address whether such rapid adaptation involved selection and sweep of the entire *EPSPS cassette* as a unit that must be fully retained versus simply a very recent origin of this cassette (thus not enough time to accumulate random mutations), more exhaustive, replicated population-level sampling and molecular evolution model-fitting analyses (including relative rate ratios of nonsynonymous and synonymous substitutions, background selection, and positive selection) need to be performed and are currently underway.

The sequences bordering the *EPSPS* gene, within the *EPSPS cassette*, contain an abundance of putative genes or gene fragments some with hallmark signatures linked to responses to stress and DNA replication and mobility. A total of 72 genes were predicted, however a majority (47) did not return a blast hit to either reference protein database (SwissProt or TrEMBL), or a positive hit with a functional domain scan. Some of these appeared to be full-length genes, including two *ricesleeper* homologs (formerly identified as an Ac transposase [15]), a reverse transcriptase, a heat shock cognate 70 kDa protein (HSC70), and a NAC domain containing protein (Additional file 2: Table S1). Expression levels in the resistant and sensitive biotypes were determined for EPSPS, the reverse transcriptase, HSC70 and the NAC domain containing protein (Table 1). These genes were transcribed in both biotypes, however it is not known if these are functional or if they are transcribed pseudogenes. If they are functional and produce protein in greater quantities than sensitive plants, as in the case of EPSPS, the basic physiology of the plant could be altered in glyphosate resistant plants. The reverse transcriptase is particularly intriguing as this type of gene is found in retrotransposons. The very high expression of this gene in the resistant biotype compared to the sensitive, where it was barely detectable, suggests that if the gene does produce a functional product, it may be relevant to the amplification mechanism. The *HSC70* and NAC domain containing protein showed variable expression between sensitive and resistant biotypes, however these are stress response genes and the plants were not subjected to any stressors at the time that RNA was extracted for gene expression analysis. Experiments in *Arabidopsis thaliana* with *HSC70-1* showed that an inducible overexpression mutant had increased heat tolerance at 44 °C [37]. However, plants with constitutive overexpression of the *HSC70-1* were difficult to obtain, indicating that an excess of this gene product may be detrimental to the plant [37]. When a NAC domain containing transcription factor was overexpressed in *Oryza sativa*, the plants showed improved drought and salt tolerance [38]. If these are functional genes, then this raises important questions regarding the response of glyphosate resistant *A. palmeri* to various abiotic stresses and whether this compounded response endows generalized and glyphosate-specific rapid adaptation. If these genes exhibit increased expression under stress conditions, this may enhance the ability of glyphosate resistant *A. palmeri* to withstand abiotic stresses, such as temperature or water/drought stress. Greater stress tolerance could influence the spread of *A. palmeri* within a changing environment.

The *ricesleeper* homolog appeared twice in the cassette, once upstream and once downstream of the *EPSPS* gene. *Ricesleeper* is a homolog of *daysleeper* in *Arabidopsis thaliana* and is considered a domesticated transposon [26] that appears to be very important during development. A TDNA insertion line in which *daysleeper* was disrupted produced slow growing, abnormal seedlings with little to no photosynthetic tissue [39]. Conversely, overexpression produced plants that grew slowly, had delayed flowering, and had poor fertility or were sterile [39]. Unfortunately for growers, glyphosate resistant *A. palmeri* exhibits none of these consequences of an overexpressed *daysleeper* homolog. Whether the *ricesleeper* homolog produces a functional product in *A. palmeri* and how that product affects plant physiology remains to be determined.

It is unknown at this point if any of the remaining genes/gene fragments are expressed in glyphosate resistant *A. palmeri*. These genes and gene fragments comprise a diverse group, including serine/threonine protein phosphatases, a phospholipase-like protein, a glutamate synthase, a ubiquitin like protease, a replication protein, a 70 kDa DNA binding subunit, and a suppressor of gene silencing 3 (SGS3). Most of these appear to be far too small to represent fully functioning genes. When BLASTp searches were performed for each of these putative genes, the amino acid product aligned to much larger homologs (data not shown). However, the *SGS3* was interesting because it was present in multiple copies – a cluster of six copies was identified about 77 kb downstream of the *EPSPS*, five of which appear to be intact in the WGS alignment of the sensitive biotype. The presence of this gene fragment six times as a cluster, may be an indication of past amplification events that were part of the evolution of the *EPSPS cassette*.

The presence of so many gene fragments may or may not be related to the amplification mechanism. Three hundred repeats were annotated in the *EPSPS cassette* (Table 2). Whether these are a result of ancient transposition events or relevant to the *EPSPS* amplification is unknown. A self-alignment reveals the complex repetitive landscape composed of interspersed DNA palindromes and simple repeats that may be configured for self-interaction through intra-molecular recombination and DNA structure formation, possibly making the *EPSPS cassette* highly dynamic. Gene fragments are signatures of transposition by helitrons and pack-MULEs [40, 41]. The cassette contains LTR, gypsy-, copia-like, LINE and SINE elements typical of retrotransposons, MULE and hAT-Ac transposons, numerous simple repeats, and helitron-like elements. As such, transposition by either a retrotransposon, or by a cut and paste mechanism, either autonomous or non-autonomous, or a rolling circle mechanism, cannot be ruled out. Until the remainder of the cassette is elucidated, it is unclear how these elements were acquired, accumulated, and proliferated randomly across the *A. palmeri* genome so quickly. It is also unclear which specific mechanism or combinations of mechanisms, if any, were involved in amplification in this resistant biotype. The abundance and structural array of elements present in a glyphosate resistant genotype suggests a rich history of concentrated transposition activity during the establishment and subsequent activities building the *EPSPS cassette*.

The presence of helitrons and their relationship to the *EPSPS cassette* build are intriguing. A putative helitron encompassed the downstream portion of the *EPSPS* coding region covering much of exon eight and also one of the putative transposase genes that immediately flank the *EPSPS* gene. This was present in both the sensitive and resistant WGS sequences, which may indicate it has an ancient origin and possibly activated or interacts with transposase gene products as part of their functional role. The helitrons also covered a MITE associated with the 5′ end of *EPSPS*. More information is needed regarding helitron localization and how helitron activity in the *EPSPS cassette* may have mediated gene rearrangements without losing expression of the functionally intact EPSPS protein.

Most interesting is the lack of contiguity of the cassette in the sensitive biotype. When the WGS reads for the sensitive biotype were mapped to the cassette sequence, over 400 gaps were present. This is in contrast to the resistant biotype in which there were no gaps and there was a much greater mapping depth, indicating amplification of the full sequence. Another feature for comparison was the number of reads in the sensitive for which the corresponding pair was missing or was improperly paired. This may imply the presence of breaks in the cassette sequence in the sensitive biotype and separate genomic locations for sections of the *EPSPS cassette* that are contiguous in the resistant biotype. The lack of contiguity was investigated and confirmed by PCR. The PCR primers designed to amplify sequences outside of the *HSC70*, *ricesleeper*, and *EPSPS* produced robust PCR products in the resistant biotype, but either failed to produce a product in the sensitive biotype or generated a much smaller product (Fig. 2). These results reinforce the bioinformatics data that show a lack of contiguity in the sensitive biotype. This may indicate a stepwise progression in assembly and amplification during the accumulated construction of the *EPSPS cassette* structural features in the resistant biotype. If the amplification resulted from periodic activation by one or more transposable elements and/or sequential orchestration amongst types of transposition and elements, what mechanism activated the series of events building the *EPSPS cassette*? Transposable elements have been known to become active in response to stress [42], and a stress response could have been part of the mechanism that led to the *EPSPS cassette*. The lack of contiguity in the sensitive biotype suggests a structurally complex evolutionary history despite the very recent and rapid origins of glyphosate resistance.

Comparative alignments with two draft genome assemblies of *Amaranthus* (*A. tuberculatus* and *A. hypochondriacus*) and the close relative *Beta vulgaris* reveal that many of the encoded genes in the *EPSPS cassette* are present in the sampled *Amaranthus* lineage, but not in *Beta vulgaris*, and that these may in fact be crucial to the segmental amplification. Moreover, lack of large scaffold N_50_ sizes and overall completeness of a representative *Amaranthus* reference genome assembly make local syntenic anslyses difficult and also justify pursuit of technologies to advance the contiguity of these assemblies into ordered pseudomolecules.

Through flow cytometry it has been established that genome size correlates with increases in the *EPSPS cassette* providing further evidence of a large segment of *EPSPS* containing DNA being preferentially amplified and distributed throughout the genome, rather than single gene amplification and dispersion in pre-existing sites in the genome. This further validates the claim that this is a novel, genome-wide mechanism of adaptive evolution in comparison to the other systems such as Kochia (14), which involves tolerance through local *EPSPS* gene copy expansions.

This investigation began as an effort to understand herbicide resistance in *A. palmeri*, but has expanded to become an exploration of movement and amplification of large DNA fragments. The size of the *EPSPS cassette* coupled with its multi-chromosomal distribution provides a unique mechanism in plant biology and provides an opportunity to study one route by which plants may rapidly evolve in response to environmental and/or abiotic (in this case herbicide) stressors. Considering that glyphosate resistance in *A. palmeri* has spread from 1 to 25 states in 10 years, this may be a means by which rapid evolution allowed the plants to survive the pressures of glyphosate exposure through either a generalized or specialized response. Thus, cassette unit construction, amplification and movement across the genome may initiate and/or facilitate rapid evolution. Until the size and end sequences of the *EPSPS cassette* are determined, and until we use larger replicated sampling of R and S populations (and species), we are limited in our understanding of how this amplification was achieved. Additional BAC sequencing will be required to expand upon the cassette. Examination of other resistant and sensitive populations will be needed to determine if this resistance evolved once or many times and how the cassette may differ, if at all, amongst recently-established versus older populations.

## Conclusions

The selection pressure imposed by glyphosate on *A. palmeri* populations has rapidly shaped the heritable herbicide resistance mechanism at the genomic level in a short period of time in this species. We have discovered a complex, multi-gene amplification unit, the *EPSPS cassette*, in *A. palmeri* that includes the *EPSPS* gene surrounded by a unique set of stress response genes as well as an array of elements and is distributed randomly throughout the genome. The complex array of repetitive elements and especially the putative helitron sequences within the *EPSPS cassette* suggest adaptive structural genomic mechanisms that drive amplification and distribution around the genome (Additional file 15: Figure S6). The high degree of amplification was consistent with measurable increases in genomic DNA content which indicates the selection pressures driven by glyphosate application may provide additional resources to promote species evolution.

## Methods

### Seed sources and plant growth conditions

Voucher specimens of *A. palmeri* from the Mississippi Delta can be found at the Mississippi Museum of Natural Science in Jackson, MS, in Stoneville’s Weed Science Laboratory’s herbarium collection. Seed used to establish plants for DNA isolation for BAC library construction and WGS were harvested from a single glyphosate resistant plant designated #13 and a single glyphosate sensitive plant designated #2. These plants were collected on the USDA research farm in Stoneville, MS. This farm is located in the approximate middle of 200-mile diameter large, flat, and open agronomic production area of cotton, corn, soybean and rice in which glyphosate is heavily used. For the flow cytometry work, to obtain DNA from a truly sensitive plant, DNA was obtained from a plant grown from seed from the Plant Introduction Station in Iowa designated as PI632235 (Additional file 16: Table S10). This seed came from Arizona in 1992 well before use of glyphosate in row crops. Plants designated as P1, P3 P4 and W3 were glyphosate resistant plants from the USDA research farm. Representative plant material is available from the author. *EPSPS* gene copy number was determined by qPCR on each of these seed lots.

Seeds of resistant *A. palmeri* were planted at 0.5-cm depth in 50-cm by 20-cm by 6-cm plastic trays with holes containing a commercial potting mix (Metro-Mix 360). Two weeks after emergence, seedlings were transplanted into 6-cm by 6-cm by 6-cm pots containing the potting mix. Trays and pots were maintained in a greenhouse set to 25/20 °C day/night, 12-h photoperiod under natural sunlight conditions supplemented with high pressure sodium lights providing 400 mmol m^−2^ s^−1^ of light intensity. Plants were fertilized biweekly with a nutrient solution (Miracle-Gro, The Scotts Company LLC, Marysville, OH, USA) containing 200 mg L^−1^ each of N, P_2_O_5_, and K_2_O 1 week after transplanting and sub-irrigated as needed, thereafter. These growth conditions were also used to generate glyphosate sensitive plants when required. When plants were 40 cm tall they were transferred to Clemson University. Prior to tissue harvesting, the seedlings were dark treated for 48 h to reduce carbohydrate synthesis and photosynthetic byproducts.

### BAC library construction, recruitment of *EPSPS* harboring BACs, and tile path assembly

All *A. palmeri* plants for BAC library construction used in this study were grown from seed obtained from a single resistant female plant. Approximately 80 g of young, expanding leaf tissue was harvested, rinsed twice in ddH_2_0, blotted dry, and immediately flash frozen in liquid nitrogen. A restriction-derived BAC library was prepared by isolating highly pure nuclei following the previously published methods of [43] with the following additions to the nuclei isolation buffer immediately before use: 1% (w/v) soluble PVP-40 (Sigma-Aldrich, St. Louis, MO), 0.1% (w/v) L-ascorbic acid (Sigma-Aldrich), 0.13% (w/v) sodium diethyldithiocarbamate trihydrate (DIECA, Sigma-Aldrich), and 0.1% beta-mercaptoethanol. To prepare high molecular weight BAC insert fragments, the nuclei-embedded agarose plugs were macerated and partially digested with *Hin*dIII for 20 min following the methods of [43]. BAC insert size selections, ligations, and transformations were performed according to published methods [43]. BAC insert sizes were determined by selecting 84 clones at random, subjecting them to alkaline lysis miniprep [44], *Not*I digestion, and standard pulsed-gel electrophoresis. The *A. palmeri* BAC library was double spotted in a 4X4 grid format on positively charged nitrocellulose membrane (GE Healthcare, Pittsburg, PA) for DNA hybridization with a Qbot (Genetix). *EPSPS* harboring BAC clones were identified in the BAC library by radiolabeling an *EPSPS*-derived PCR amplicon using the DECAprime II DNA Labeling Kit (Thermo Fisher, Waltham, MA) following the manufacturers recommended instructions. The probe was developed by amplifying a 198 bp segment of *EPSPS* spanning exons 4 and 5. The Takara LA PCR kit v2.1 (Clontech, Mountain View, CA) was used to amplify the probe. Reactions consisted of ~50 ng DNA, 100 nM primers, 2.5 mM MgCl_2_, 400 μM dNTPs, 1 x buffer, 2.5 units of polymerase and H_2_O to 50 μL. Cycle conditions were 94 °C 1 min, 30 cycles of 90 °C for 30s, 55 °C for 30s, and 72 °C for 1 min, and 72 °C for 5 min. The PCR product was purified by agarose gel extraction using the GenElute^TM^ gel extraction kit (Sigma Aldrich). BAC hybridizations were performed following previously published methods [45]. High Information Content Fingerprinting (HICF) [46] was performed on positively identified BAC clones to establish an overlapping tile path. BAC DNA was purified following standard alkaline lysis miniprep methods [44]. Purified BAC DNA was digested with *Ban*I, *Hin*dIII, *Nhe*I, *Xho*I, and *Pvu*II and labeled with the SNaP-shot labeling kit (Life Technologies, Carlsbad, CA) following the procedures of [47]. Prior to capillary electrophoresis, 9 μL of Hi-Di formamide (Life Technologies) and 0.05 μL of LIZ1200 size standard (Life Technologies) were added to each well. BAC restriction profiles were resolved on an ABI3730xl (Life Technologies) with a 50 cm array. The raw data were processed for sizing quality with the GeneMapper software package (Life Technologies) and converted to digital fingerprints with FPMiner (Bioinforsoft, Beaverton, OR). BAC fingerprints were filtered for vector bands and clones with less than 20 or more than 200 bands. Preprocessed data were uploaded to FPC v8.5.3 [48] for contig assembly. A *de novo* fingerprint assembly was performed with a Sulston cutoff of 1e^−50^ and tolerance setting of 3.0. Questionable clones (Q-clones) were removed with the DQ function using a setting of 10% and an iteration of contig end merges was performed with the Ends-To-Ends function with a Sulston cutoff of 1e-35 with contig merge requirements of at least 20 consensus band (CB) matches and overlap with at least 2 end clones. An iteration of Singles-To-Ends was performed to incorporate singletons to contigs with a Sulston cutoff of 1e-35, and another round of DQ’ing was performed at 10%. Overgo probes for extension of the cassette using the OvergoMaker software (http://bioinf.wehi.edu.au/cgi-bin/overgomaker) and hybridizations performed following the methods of [49]. A minimal tile path (MTP) of representative BAC clones was selected manually. These clones were subject to BAC-end sequencing by dye-terminator sequencing (BigDye version 3.1; Life Technologies) of the clone ends using the universal priming sites T7 and M13 reverse that flank the multi-cloning site of the BAC vector.

### BAC sequencing, assembly, and annotation

BAC insert sequencing was performed using two different shotgun-style approaches, Illumina and Pacific Biosciences. Low host containing BAC DNA was prepared by growing each BAC individually as a 45 mL culture (2XYT media, Thermo Fisher) following procedures of [50]. Individually indexed Illumina DNA sequencing libraries were performed with the Nextera XT kit (Illumina, San Diego, CA) for each BAC and sequenced on a single channel of an Illumina MiSeq using a 2x250bp PE sequencing protocol. Assemblies were attempted with Phrap [51] and Abyss [52] which resulted in hundreds to thousands of very short contigs. This approach was supplanted with Pacific Biosciences RSII+ single molecule reads by pooling clone DNA in equimolar ratios and collecting a single Single-Molecule real-Time Sequencing (SMrT) cell (P6-C4 chemistry) for each pool of BAC templates (first and second round), respectively. Raw sequence data was filtered for short reads (<1000 bp), error corrected using the Self-Correction module of PBcR, and the longest 25X representative coverage of reads used for assembly with the Celera CA assembler v8.2 [53]. The resulting consensus was polished with Quiver (https://github.com/PacificBiosciences/GenomicConsensus). Putative gene sequences were predicted with the MakerP software v.2.28 (http://www.yandell-lab.org/software/maker.html) and deployed on the iPlant Cyberinftrastructure (NSF ISO-1126998). Predicted coding sequences were annotated for homology by BLASTX alignment to the non-redundant protein database in GenBank, and functional signatures with the Interproscan v5.7-48 tool [54]. Repetitive and low complexity sequence was identified with the RepeatMasker software v4.0.6 [55]. Putative helitron sequences were predicted with the HelitronScanner software [56] with an LCV score of 3 for both the Head and Tail scans. A self-alignment of the reference *EPSPS cassette* sequence was performed with the nucmer module of the Mummer v3.22 software [57]. Alignment coordinates were extracted and plotted as links with the Circos software [58].

### Whole genome shotgun sequencing and comparison of resistant and susceptible biotypes

Whole genome shotgun sequences for a resistant and susceptible biotype were collected by purifying whole genome DNA following standard CTAB plant DNA extraction procedures. An Illumina sequencing library was prepared for each biotype following standard TruSeq protocols (Illumina) and sequenced on a single lane of HiSeq2500 using a 2x125bp paired-end read type. Raw sequencing data was preprocessed to remove reads less than 36 bp and low quality bases with the Trimmomatic software [59]. Preprocessed reads were aligned to the *A. palmeri EPSPS* reference cassette sequencing using the Bowtie2 short read aligner [60]. Per base coverage relative the reference cassette sequence was determined with Samtools [61] and plotted in circular format with Circos [58].

### qPCR analysis

RNA was extracted from sensitive and resistant *A. palmeri* using the RNEasy plant mini kit (Qiagen, Valencia, CA). Leaf tissue was homogenized in the extraction buffer using a mortar and pestle. Following extraction and purification, RNA was DNAse treated. Reactions consisted of the RNA, 1X RDD buffer, and 3 units of DNAse (Qiagen). The reactions were incubated at room temperature for 30 min and then purified on a RNEasy plant mini kit column. The quality and quantity of RNA were assessed by an A_260_ reading, A_260/280_ ratio, and agarose gel electrophoresis. cDNA was generated using the high capacity cDNA reverse transcription kit (Life Technologies, Grand Island, NY). Reactions consisted of 2 μg RNA, 1 X RT buffer, 4 mM dNTPs, 1 X random primers, 50 U reverse transcriptase, and H_2_O to 20 μL. Cycle conditions were 25 °C for 10 min, 37 °C for 2 h, 85 °C for 5 min, and a 4 °C hold. qPCR was performed to determine expression levels for *EPSPS*, *HSC70*, a NAC domain containing protein and a reverse transcriptase with primer pairs AW146 and AW147, AW550 and AW551, AW556 and AW557, and AW548 and AW549 (see Additional file 9: Table S7 for primer sequences). ALS was used as a reference (primers AW23 and AW24, from [5]). Reactions consisted of 25 ng cDNA, 1.5 μM primers, 1 X Power Sybr Green master mix (Life Technologies), and H_2_O to 50 μL. Reactions were performed using an ABI 7500 Real Time PCR System. Cycle conditions are as follows: 50 °C for 2 min, 95 °C for 10 min, 40 cycles of 95 °C for 15 s and 60 °C for 1 min, and a 4 °C hold. A melt curve analysis was included. Copy number assays for *EPSPS* were similar to the gene expression assays, except that 10 ng of genomic DNA was used in place of RNA. Data were analyzed according to the standard curve method and error was calculated as described in ABI User Bulletin #2 (Life Technologies).

### PCR analysis of cassette

DNA was extracted from leaf tissue of resistant and sensitive plants by homogenization with a mortar and pestle in an extract buffer described by Paterson et al. [62]. The homogenate was incubated at 65 °C for 1 h and then purified on a DNEasy plant mini column (Qiagen). Quality and quantity were assessed by an A_260_ reading, A_260/280_ ratio, and agarose gel electrophoresis. For PCR the following primer pairs were used: AW550 and AW553, AW502 and AW293, AW426 and AW511, AW176 and AW211, AW301 and AW168, AW524 and AW129, AW128 and AW100, AW156 and AW533, and AW544 and AW259 (Additional file 9: Table S7). Reactions consisted of ~ 50 ng DNA, 0.625 μM primers, 1 X Thermo Scientific^TM^ Maxima Hot Start Green PCR Master Mix (Fisher Scientific), and H_2_O to 20 μL. Cycle conditions were as follows: 94 °C for 4 min, 30 cycles of 94 °C for 30 s, 55 °C for 30 s, and 72 °C for 1 min 30 s, 72 °C for 5 min and a 4 °C hold. PCR products were analyzed by agarose gel electrophoresis.

### Syntenic alignments and flow cytometry

Whole genome alignments were performed with the promer wrapper scripts and filtered for matches with 60% identity and match length of 100 bp, as part of the Mummer alignment software v3.0 [63, 64]. Dot plots were generated with the MummerPlot wrapper scripts. The procedure used to analyze nuclear DNA content in plant cells was modified from Arumuganathan and Earle [65]. Briefly, the procedure consists of preparing suspensions of intact nuclei by chopping of 50 mg plant tissues in MgSO_4_ buffer mixed with DNA standards and stained with DNAase/ RNAase free propidium iodide (PI) solution. Fluorescence intensities of the stained nuclei were measured by a FACScalibur flow cytometer (Becton-Dickinson, San Jose, CA). Values for nuclear DNA content were estimated by comparing fluorescence intensities of the nuclei of the test population with those of an appropriate internal DNA standard that is included with the tissue being tested. We used nuclei from Chicken Red blood cells (2.5 pg/2C), *Glycine max* (2.45 pg. /2C), *Oryza sativa* cv Nipponbare (0.96 pg/ 2C), or *Arabidopsis thaliana* (0.36 pg/2C) as the internal standard. The pellet was suspended by vortexing vigorously in 0.5 mL solution containing 10 mM MgSO_4_.7H_2_O, 50 mM KCl, 5 mM Hepes, pH 8.0, 3 mM dithiothreitol, 0.1 mg / mL propidium iodide, 1.5 mg/mL DNAse free RNAse (Rhoche, Indionapolis, IN) and 0.25% Triton X-100. The suspended nuclei were withdrawn using a pipettor, filtered through 30-μm nylon mesh, and incubated at 37 °C for 30 min before flow cytometric analysis. Suspensions of sample nuclei was spiked with suspension of standard nuclei (prepared in above solution) and analyzed with a FACScalibur flow cytometer (Becton-Dickinson, San Jose, CA). For each measurement, the propidium iodide fluorescence area signals (FL2-A) from 1000 nuclei were collected and analyzed by CellQuest software (Becton-Dickinson, San Jose, CA). The mean position of the G0/G1 (Nuclei) peak of the sample and the internal standard were determined by CellQuest software. The mean nuclear DNA content of each plant sample, measured in picograms, was based on 1000 scanned nuclei.

## Additional files

Additional file 1: Figure S1. A) Autorad images of hybridizing the *A. palmeri* BAC library with the *EPSPS* gene sequence. B) Autorad images of hybridizing the BAC library with overgo probes designed from terminal sequences from the first 5 clone BAC pool assembly to identify extension BACs. (PDF 17765 kb)
Additional file 2: Table S1. Gene annotation of the EPSPS cassette. (XLSX 34 kb)
Additional file 3: Table S2. EPSPS gene copy number determination by qRT-PCR. (XLSX 8 kb)
Additional file 4: Table S3. Repeat characterization of the EPSPS cassette. (XLSX 20 kb)
Additional file 5: Figure S2. The *EPSPS cassette* self-alignment. Red dots indicate direct alignment, and blue are inverted. Palindromic block arrays flank the *EPSPS* locus, with larger inverted repeats surrounding 2 of the palindromic blocks. (PDF 3948 kb)
Additional file 6: Table S4. Predicted helitrons in the EPSPS amplicon with an LCV score of at least 3. (XLSX 8 kb)
Additional file 7: Table S5. Read mapping summary for the resistant and sensitive WGS. (XLSX 8 kb)
Additional file 8: Table S6. Alignment of the resistant (R) and sensitive (S) whole genome shotgun reads to gene sequences located distal to the EPSPS genomic interval. (XLSX 9 kb)
Additional file 9
**Table S7**. Predicted helitron depth in R and S wgsalignment to EPSPS cassette sequence. (XLSX 9 kb)
Additional file 10: Figure S3. SNP track (red and blue stacked glyphs) for the S and R biotypes. The inner track (red) highlights SNPs that are unique and heterozygous between the S and the *EPSPS* reference interval. The subsequent track (blue) are SNPs that highlight unique alternate alleles (genotypes) not found in alternate biotype or reference. (PDF 10920 kb)
Additional file 11: Figure S4. A. Alignment of the EPSPS cassette to *A. tuberculatus* draft genome assembly; B. the draft *A. hypochondriacus* assembly; and C. *B. vulgaris*. Alignments were restricted to 60% identity and match length of 100 bp. (PDF 6565 kb)
Additional file 12: Table S8. Primers used in polymerase chain reactions. (XLSX 9 kb)
Additional file 13: Figure S5. A. Alignment of the *EPSPS cassette* genes to the *A. hypochondriacus* draft genome assembly that illustrates colinearity with many of the EPSPS predicted genes; B. Alignment of the EPSPS cassette with the EPSPS containing scaffold (kn127846.1) of A. hypochondriacus; the exons of the *EPSPS* gene are highlighted in grey. (PDF 4782 kb)
Additional file 14: Table S9. Structural comparison between the EPSPS cassette and *A. tuberculatus*, and *B. vulgaris*. (XLSX 8 kb)
Additional file 15: Figure S6. A. Alignment of the *EPSPS cassette* genes to the *B. vulgaris* genome that illustrates colinearity of the reverse transcriptase, heat-shock, *EPSPS*, *SGS3*, and NAC domain containing genes. B. Alignment of the *EPSPS cassette* with the *EPSPS* containing scaffold (KI865360.1) of beta vulgaris; the exons of the *EPSPS* gene are highlighted in grey. (PDF 3157 kb)
Additional file 16: Table S10. Flow cytometry genome sizing data for 5 genotypes that indicate significant differences in size that correlates with EPSPS gene copy number. (XLSX 10 kb)

## Abbreviations

BAC: Bacterial artificial chromosome
EPSPS: 5-enolpyruvylshikimate-3-phosphate synthase
HICF: High information content fingerprinting
PCR: Polymerase chain reaction
qPCR: Quantitative polymerase chain reaction
WGS: Whole genome shotgun sequencing
