# Supplementary figures and images for: The unique genomic landscape surrounding the EPSPS gene in glyphosate resistant Amaranthus palmeri: a repetitive path to resistance
Source: BMC Genomics. 2017 Jan 17;18:91. doi: 10.1186/s12864-016-3336-4 (PMC5240378; doi:10.1186/s12864-016-3336-4)

A.

Filter A

Filter B

*EPSPS* Hybridization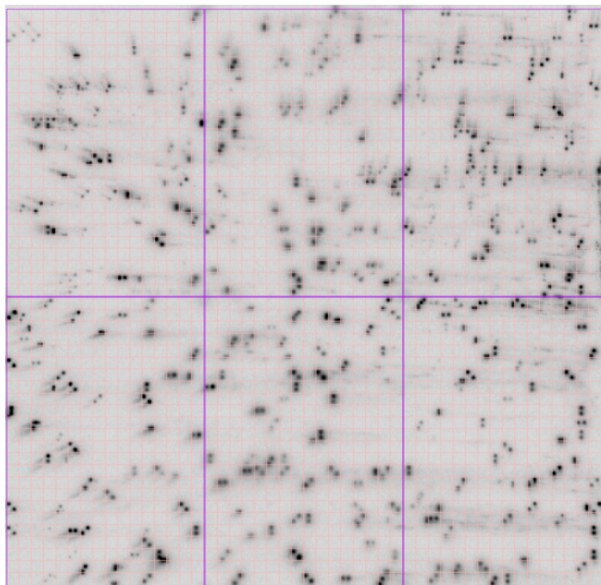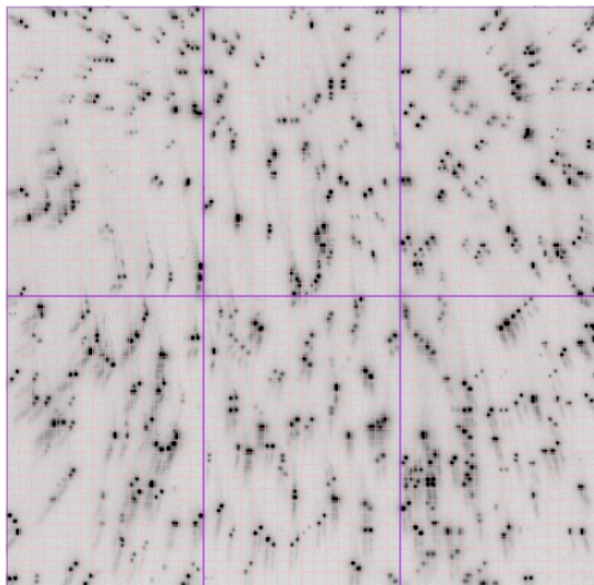

B.

Extension Hybridization

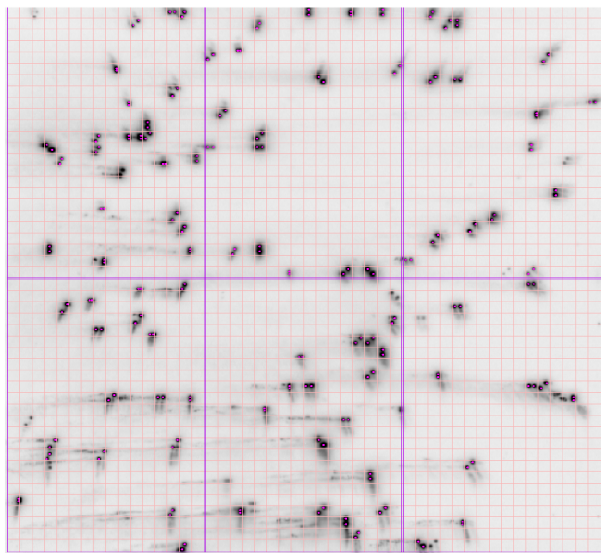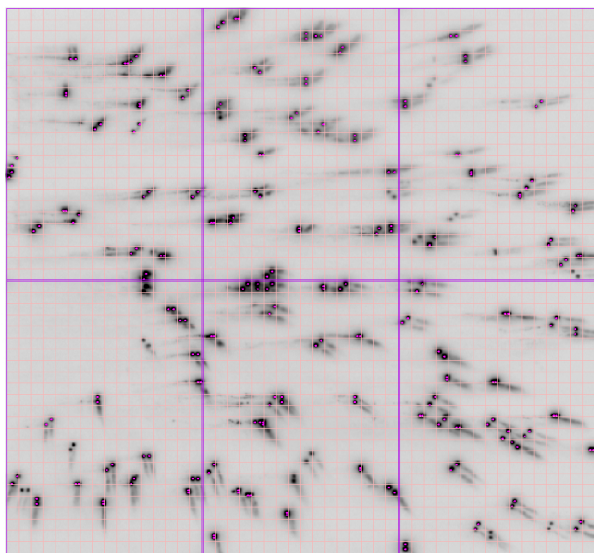

Supplement: Additional file 1: Figure S1. — A) Autorad images of hybridizing the A. palmeri BAC library with the EPSPS gene sequence. B) Autorad images of hybridizing the BAC library with overgo probes designed from terminal sequences from the first 5 clone BAC pool assembly to identify extension BACs. (PDF 17765 kb) [file 12864_2016_3336_MOESM1_ESM.pdf]

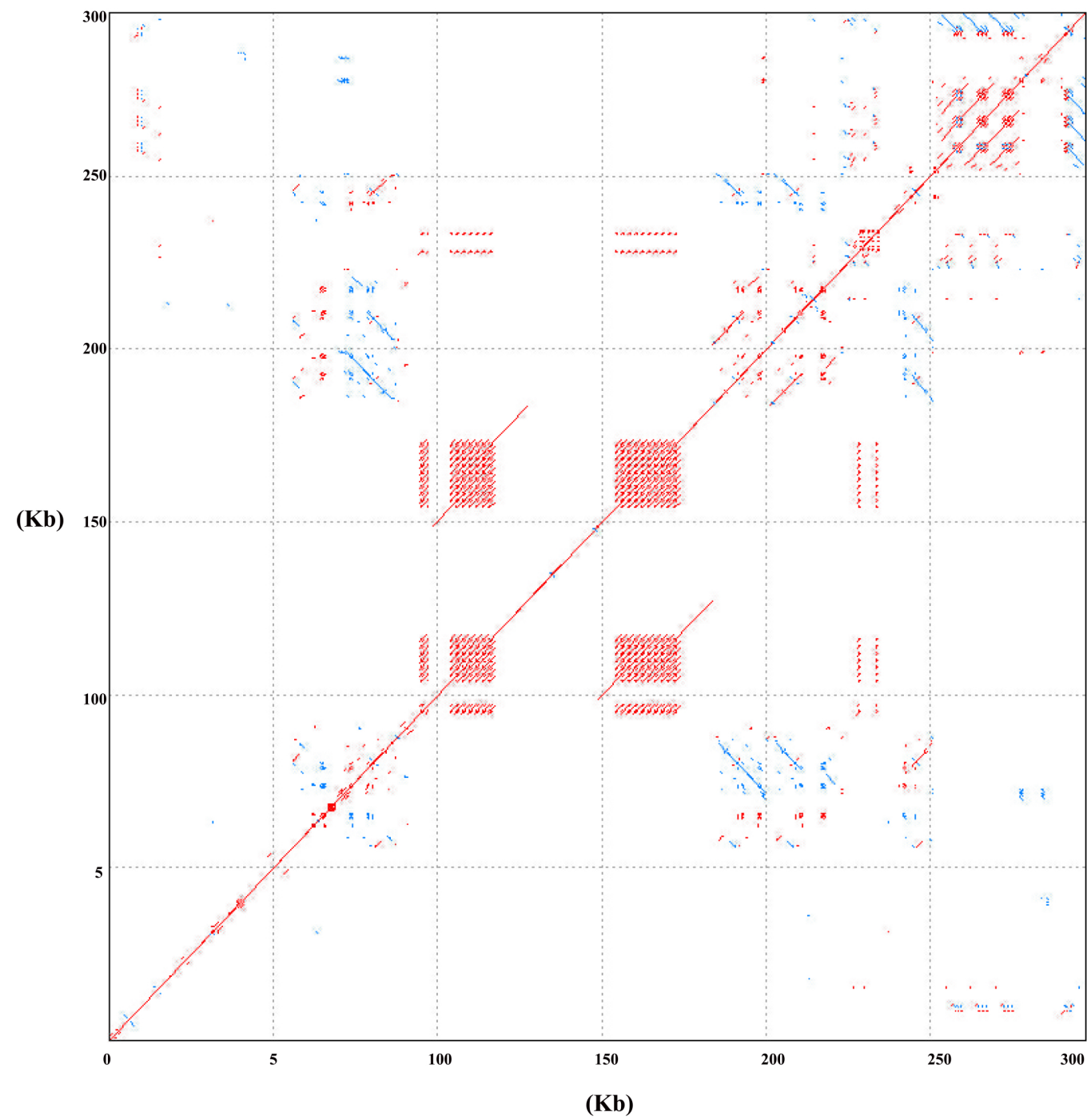

Supplement: Additional file 5: Figure S2. — The EPSPS cassette self-alignment. Red dots indicate direct alignment, and blue are inverted. Palindromic block arrays flank the EPSPS locus, with larger inverted repeats surrounding 2 of the palindromic blocks. (PDF 3948 kb) [file 12864_2016_3336_MOESM5_ESM.pdf]

Glyphosate sensitive

Glyphosate resistant

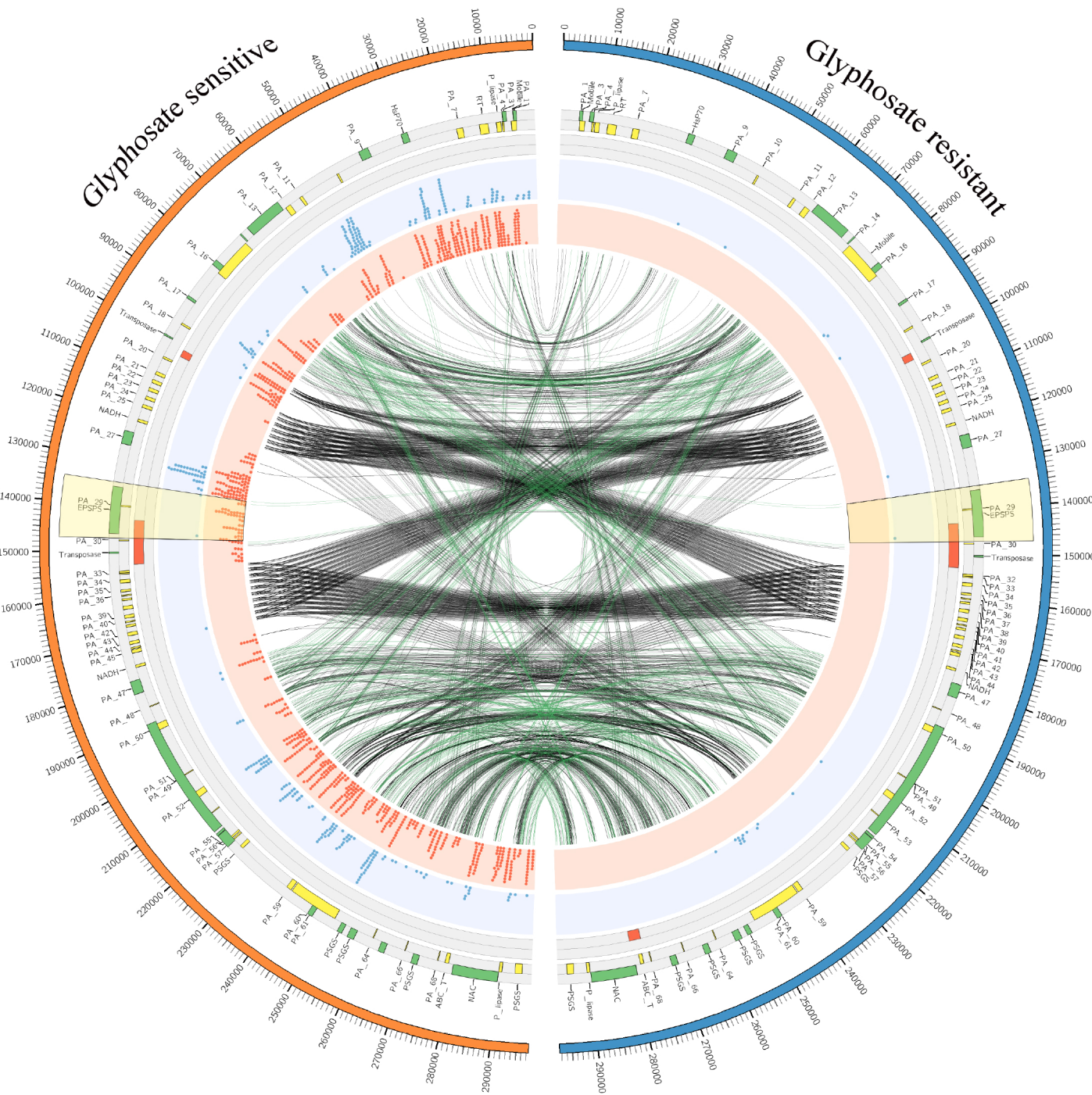

Supplement: Additional file 10: Figure S3. — SNP track (red and blue stacked glyphs) for the S and R biotypes. The inner track (red) highlights SNPs that are unique and heterozygous between the S and the EPSPS reference interval. The subsequent track (blue) are SNPs that highlight unique alternate alleles (genotypes) not found in alternate biotype or reference. (PDF 10920 kb) [file 12864_2016_3336_MOESM10_ESM.pdf]

A.

*A. hypochondriacus*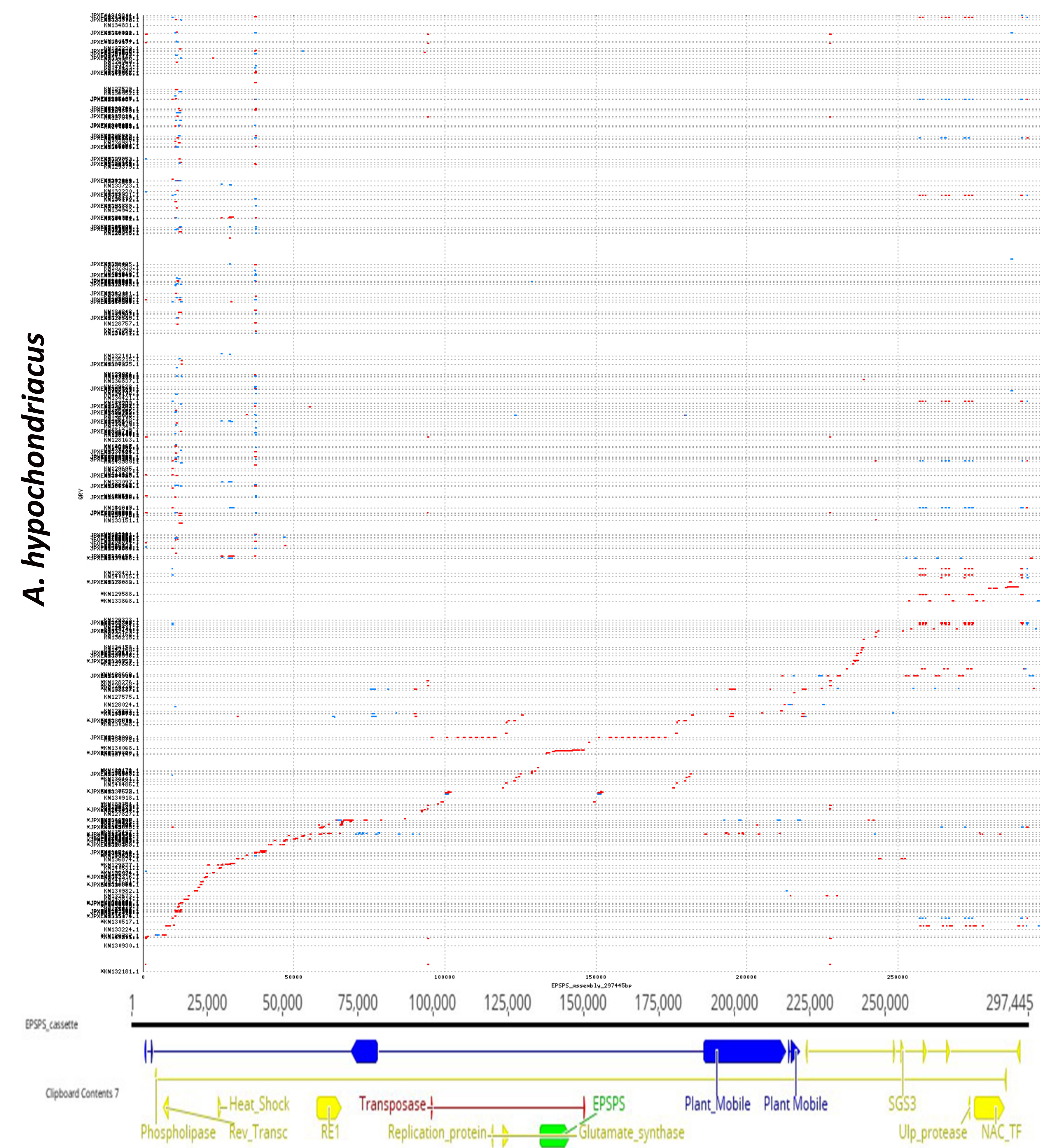

C.

*B. vulgaris*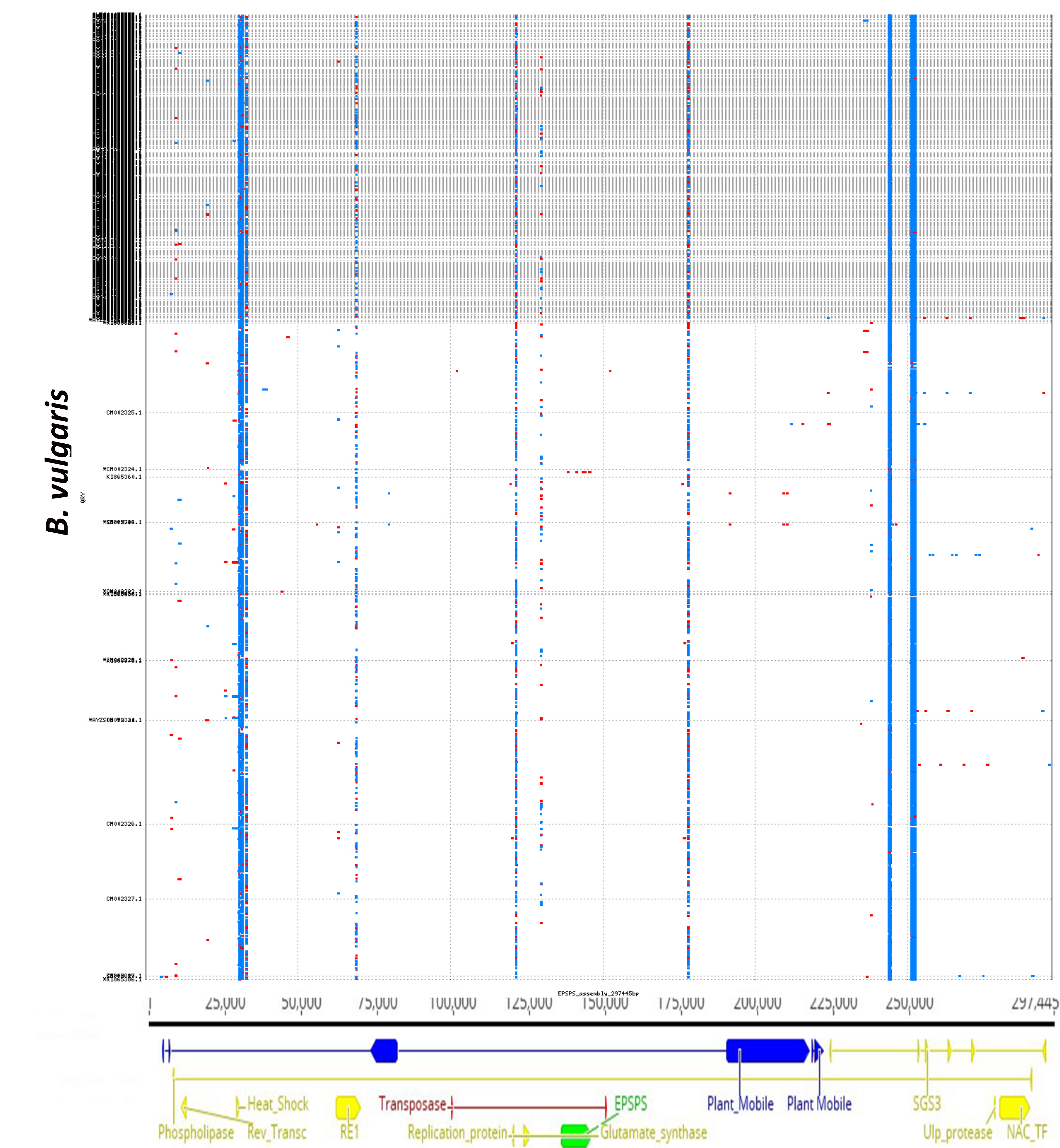

B.

*A. tuberculatus*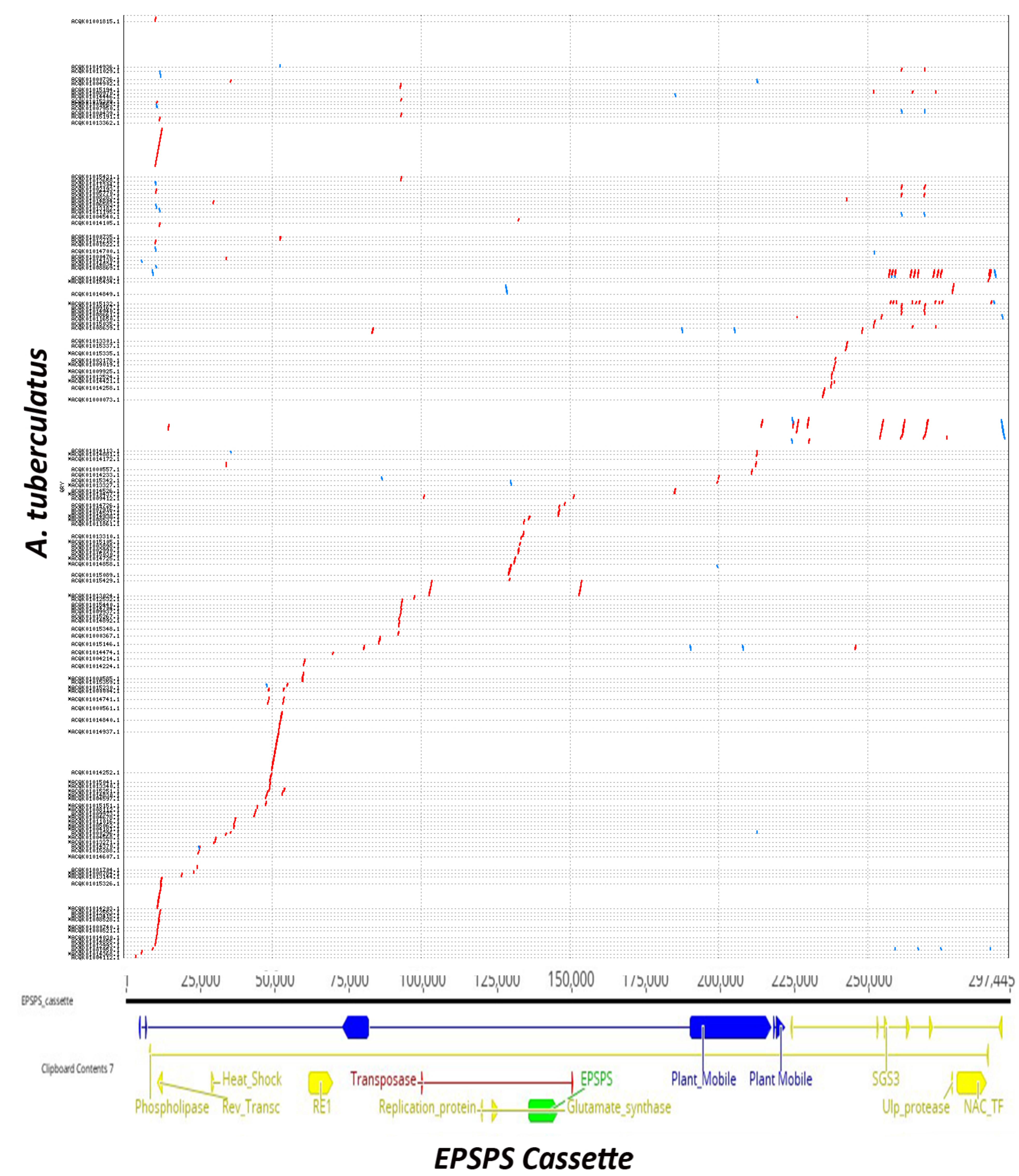

Supplement: Additional file 11: Figure S4. — A. Alignment of the EPSPS cassette to A. tuberculatus draft genome assembly; B. the draft A. hypochondriacus assembly; and C. B. vulgaris. Alignments were restricted to 60% identity and match length of 100 bp. (PDF 6565 kb) [file 12864_2016_3336_MOESM11_ESM.pdf]

A.

*A. hypochondriacus*

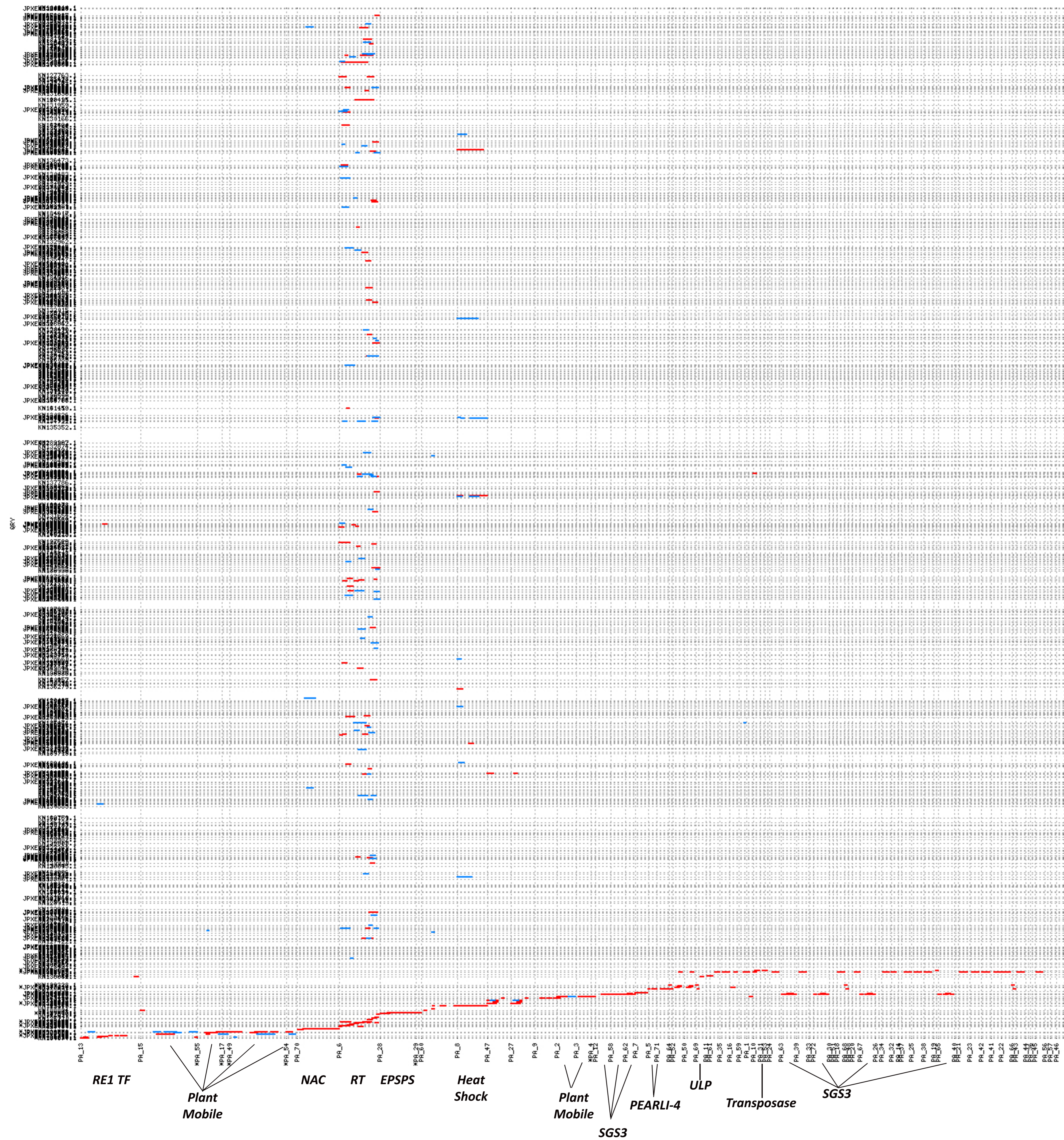

**EPSPS Cassette Genes**

B.

*A. hypochondriacus (kn127846.1)*

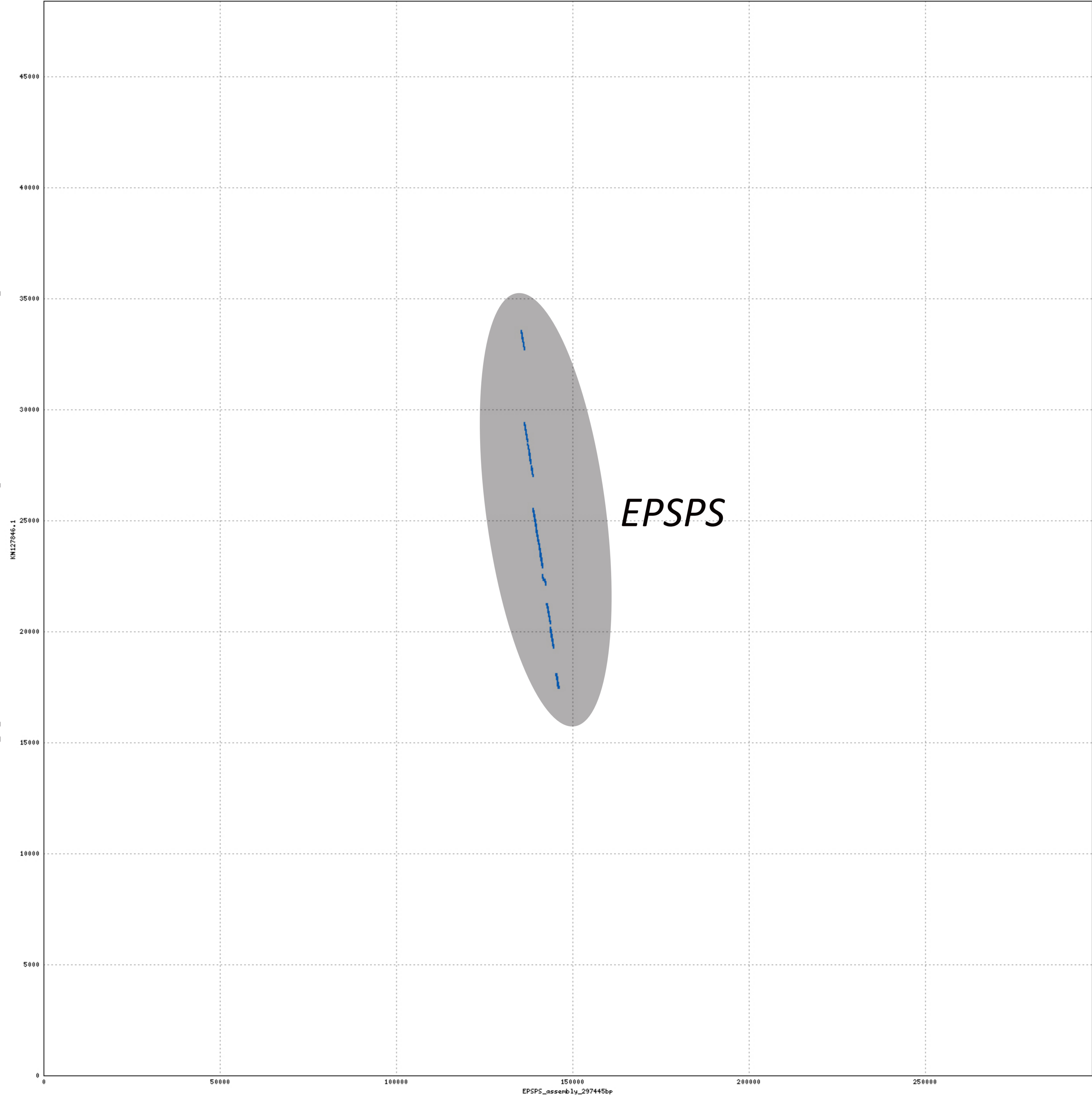

**EPSPS Cassette**

Supplement: Additional file 13: Figure S5. — A. Alignment of the EPSPS cassette genes to the A. hypochondriacus draft genome assembly that illustrates colinearity with many of the EPSPS predicted genes; B. Alignment of the EPSPS cassette with the EPSPS containing scaffold (kn127846.1) of A. hypochondriacus; the exons of the EPSPS gene are highlighted in grey. (PDF 4782 kb) [file 12864_2016_3336_MOESM13_ESM.pdf]

A.

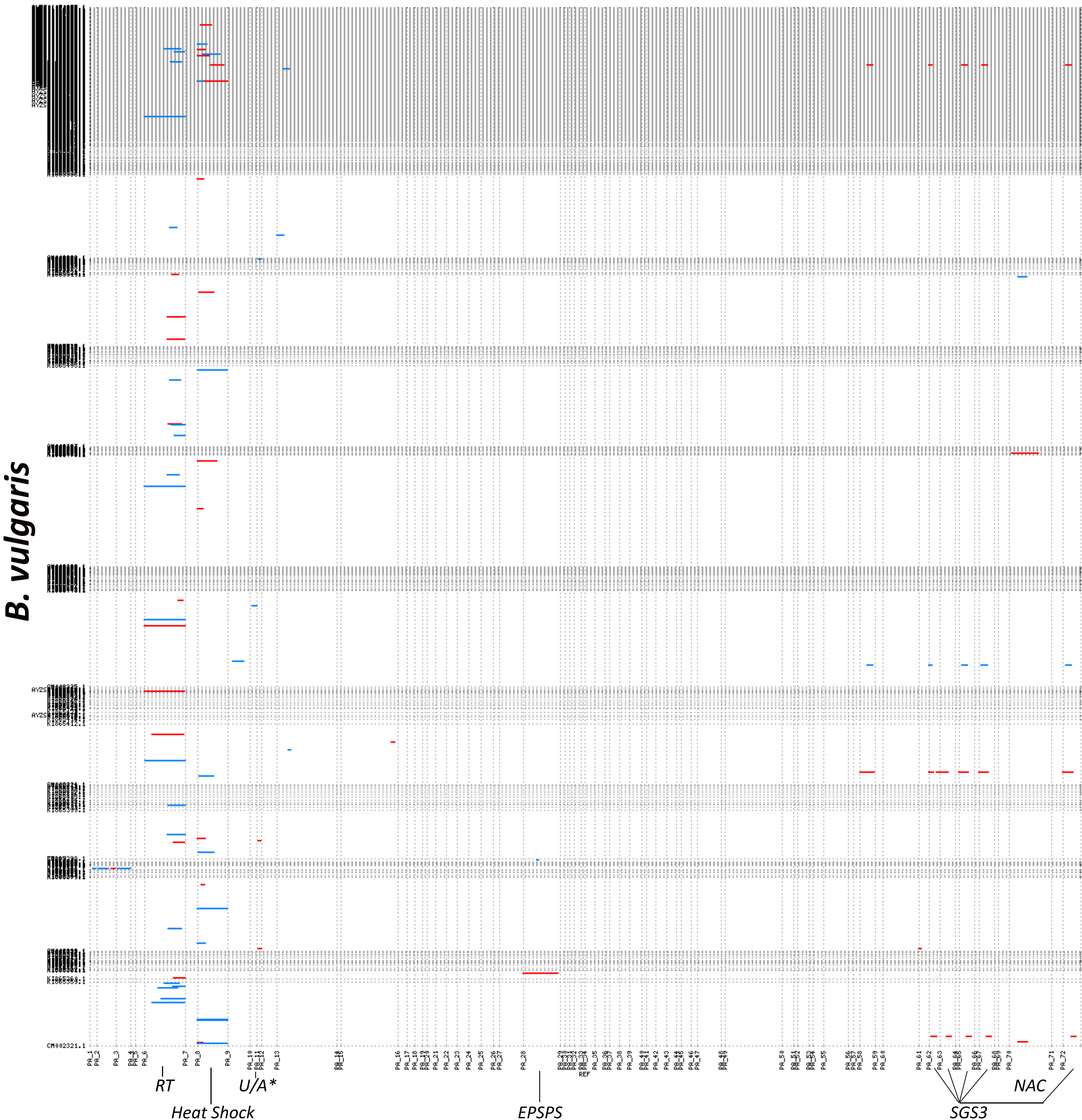

**EPSPS Cassette Genes**

B.

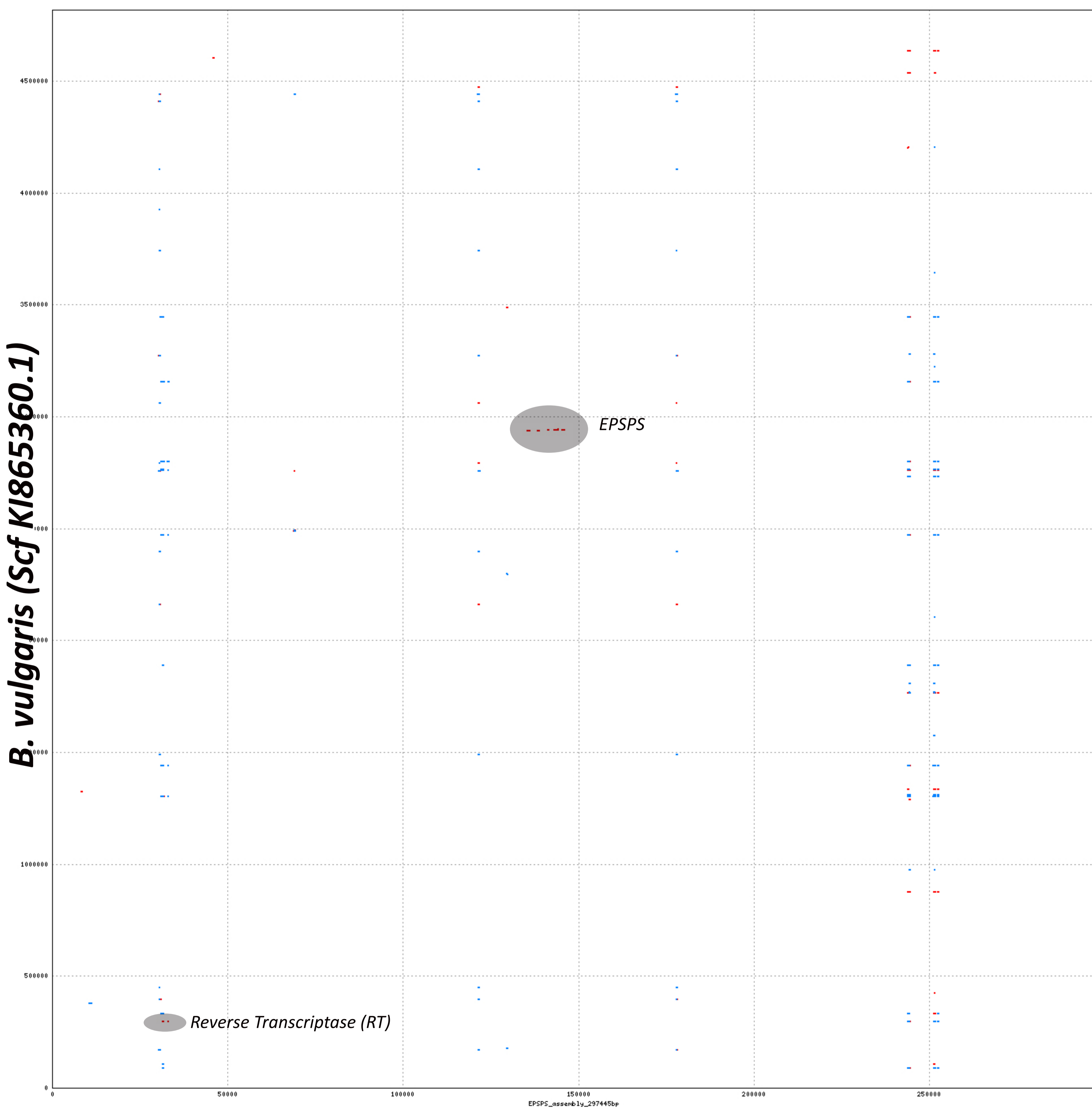

**EPSPS Cassette**

Supplement: Additional file 15: Figure S6. — A. Alignment of the EPSPS cassette genes to the B. vulgaris genome that illustrates colinearity of the reverse transcriptase, heat-shock, EPSPS, SGS3, and NAC domain containing genes. B. Alignment of the EPSPS cassette with the EPSPS containing scaffold (KI865360.1) of beta vulgaris; the exons of the EPSPS gene are highlighted in grey. (PDF 3157 kb) [file 12864_2016_3336_MOESM15_ESM.pdf]
